# Integrative physiology and transcriptome reveal salt-tolerance differences between two licorice species: Ion transport, Casparian strip formation and flavonoids biosynthesis

**DOI:** 10.1186/s12870-024-04911-1

**Published:** 2024-04-11

**Authors:** Xin Li, Ying Xu, Jiade Zhang, Ke Xu, Xuerong Zheng, Jiafen Luo, Jiahui Lu

**Affiliations:** College of Life Sciences, Shihezi University, Shihezi, 832003 China

**Keywords:** Medicinal plants, Licorice, Salt stress, Casparian strip, Flavonoids

## Abstract

**Background:**

*Glycyrrhiza inflata* Bat. and *Glycyrrhiza uralensis* Fisch. are both original plants of ‘Gan Cao’ in the *Chinese Pharmacopoeia*, and *G. uralensis* is currently the mainstream variety of licorice and has a long history of use in traditional Chinese medicine. Both of these species have shown some degree of tolerance to salinity, *G. inflata* exhibits higher salt tolerance than *G. uralensis* and can grow on saline meadow soils and crusty saline soils. However, the regulatory mechanism responsible for the differences in salt tolerance between different licorice species is unclear. Due to land area-related limitations, the excavation and cultivation of licorice varieties in saline-alkaline areas that both exhibit tolerance to salt and contain highly efficient active substances are needed. The systematic identification of the key genes and pathways associated with the differences in salt tolerance between these two licorice species will be beneficial for cultivating high-quality salt-tolerant licorice *G. uralensis* plant varieties and for the long-term development of the licorice industry. In this research, the differences in growth response indicators, ion accumulation, and transcription expression between the two licorice species were analyzed.

**Results:**

This research included a comprehensive comparison of growth response indicators, including biomass, malondialdehyde (MDA) levels, and total flavonoids content, between two distinct licorice species and an analysis of their ion content and transcriptome expression. In contrast to the result found for *G. uralensis*, the salt treatment of *G. inflata* ensured the stable accumulation of biomass and total flavonoids at 0.5 d, 15 d, and 30 d and the restriction of Na^+^ to the roots while allowing for more K^+^ and Ca^2+^ accumulation. Notably, despite the increase in the Na^+^ concentration in the roots, the MDA concentration remained low. Transcriptome analysis revealed that the regulatory effects of growth and ion transport on the two licorice species were strongly correlated with the following pathways and relevant DEGs: the TCA cycle, the pentose phosphate pathway, and the photosynthetic carbon fixation pathway involved in carbon metabolism; Casparian strip formation (lignin oxidation and translocation, suberin formation) in response to Na^+^; K^+^ and Ca^2+^ translocation, organic solute synthesis (arginine, polyamines, GABA) in response to osmotic stresses; and the biosynthesis of the nonenzymatic antioxidants carotenoids and flavonoids in response to antioxidant stress. Furthermore, the differential expression of the DEGs related to ABA signaling in hormone transduction and the regulation of transcription factors such as the HSF and GRAS families may be associated with the remarkable salt tolerance of *G. inflata*.

**Conclusion:**

Compared with *G. uralensis*, *G. inflata* exhibits greater salt tolerance, which is primarily attributable to factors related to carbon metabolism, endodermal barrier formation and development, K^+^ and Ca^2+^ transport, biosynthesis of carotenoids and flavonoids, and regulation of signal transduction pathways and salt-responsive transcription factors. The formation of the Casparian strip, especially the transport and oxidation of lignin precursors, is likely the primary reason for the markedly higher amount of Na^+^ in the roots of *G. inflata* than in those of *G. uralensis*. The tendency of *G. inflata* to maintain low MDA levels in its roots under such conditions is closely related to the biosynthesis of flavonoids and carotenoids and the maintenance of the osmotic balance in roots by the absorption of more K^+^ and Ca^2+^ to meet growth needs. These findings may provide new insights for developing and cultivating *G. uralensis* plant species selected for cultivation in saline environments or soils managed through agronomic practices that involve the use of water with a high salt content.

**Supplementary Information:**

The online version contains supplementary material available at 10.1186/s12870-024-04911-1.

## Background

The leguminous licorice plant *Glycyrrhiza inflata* Bat. and *Glycyrrhiza uralensis* Fisch. are the key plants listed under the name ‘Gan Cao’ in the *Chinese Pharmacopoeia* [1]. Due to its high medicinal potential, *G. uralensis* is currently the mainstream variety of licorice and has a long history of use in traditional Chinese medicine. The notable anti-inflammatory and immunomodulatory effects of *G. uralensis*, coupled with accumulated practical experience, have elevated its popularity in traditional medical practices [1, 2]. *G. uralensis* has shown some degree of tolerance to salinity, but a high salt environment can nevertheless limit its growth and affect its yield and quality [3–5]. Studies have shown that *G. inflata* exhibits higher salt tolerance than *G. uralensis* and that *G. inflata* can grow on saline meadow soils and even crusty saline soils [5, 6]. However, this difference in salt tolerance has been revealed only physiologically, and the exact underlying mechanisms have not yet been elucidated [4, 7]. Due to dwindling wild medicinal licorice resources, the development and cultivation of licorice are vital in traditional medicine. Due to the land area-related limitations, the excavation and cultivation of licorice varieties in saline-alkaline areas both exhibit tolerance to salt and contain highly efficient active substances [6–8]. Currently, relatively few plants are under industrial development for the purpose of selecting those with high tolerance to salt, and the majority of plants grown on saline soil have been selected for food or ornamental purposes [9]. In this context, the systematic discovery of the key genes and pathways involved in the difference in salt tolerance between the two licorice species will be beneficial for cultivating high-quality salt-tolerant licorice *G. uralensis* plant varieties, repairing saline-alkaline soil, and achieving long-term development in the licorice industry.

An excessive sodium chloride concentration in soil can significantly contribute to elevated salinity, thereby negatively affecting plant productivity, and causing an imbalance in permeability homeostasis and nutrient loss [10, 11]. Self-healing mechanisms, including those that control the absorption of sodium ions, the transport of mineral elements, the accumulation of organic solutes, and the improvement of antioxidant function, can be used by plants to resist salt damage [3, 12, 13]. In primary metabolism, plants can exploit substrates needed for biosynthesis by promoting energy (central carbon) metabolism and transferring energy from basal metabolism to stress adaptation [5, 14]. Nevertheless, the antioxidant capacity of the root system of *G. inflata* may be markedly stronger than that of *G. uralensis* [15, 16]. Salt-tolerant plants have been found to accumulate more Na^+^ in roots, reducing the Na^+^ content in the aboveground parts and thereby promoting plant growth [17]. A previous study showed that the sodium/hydrogen exchanger (NHX7) gene on the plasma membrane can participate in Na^+^ efflux to the extracellular space, whereas the antiporter NHX2 gene mediates Na^+^ transport to vacuoles, thereby relieving Na^+^ damage to the cell [12, 17, 18]. Moreover, Na^+^ can be blocked from entering through the stele to the upper part of the plant through regulation of the formation of the endodermis barrier, which contains apoplastic (Casparian strip, CS) and transcellular (suberin lamellae) barriers [12]. The CS is a band of woody and corybantic structures located in the inner cortex of the root that acts as an exocytoplasmic barrier to harmful ions in the root, preventing their passage through the central column [6]. Both mechanisms help confine Na^+^ within specific regions of the root system, thereby alleviating the adverse effects on plant growth and development. The major strategy used by plants to cope with osmotic disorders involves increasing the absorption of inorganic ions from the environment. In the realm of plant stress resistance, inorganic ions, such as K^+^, Ca^2+^, and Mg^2+^, can not only participate in a range of metabolic pathways but also maintain a relatively stable osmotic potential in cells [17, 19]. In addition, plants employ osmotic regulation to sequester ions into vacuoles, preventing their interference with enzymes in the cytosol. To maintain the water potential balance within the cell during the process, other solutes, such as betaines and polyamines, may accumulate in the cytosol, increasing the solute concentration and reducing the cell water potential, which enhances the plant’s water-absorbing capacity [20]. Enzymatic and nonenzymatic defenses are the two main systems for eliminating peroxide [21, 22]. The enzyme system helps mitigate stress-induced damage by removing reactive oxygen species (ROS). However, when the cellular ROS production exceeds the capacity of the enzyme system, relying solely on this process may not be enough to shield the cell from oxidative stress. Consequently, cells frequently rely on the coordinated control of antioxidants to ensure their protection [20]. Related substances from another system, carotenoids, and flavonoids, can also react with peroxidation products or directly scavenge ROS to alleviate oxidative stress [23]. Under salt stress, the endogenous hormones of plants potentially promote and regulate the stress response. An increase in the ABA levels under salt and alkali stress promotes root elongation to result in more effective absorption of water and nutrients. Ethylene can activate a series of antioxidant enzymes, increasing the levels of antioxidant substances, which play an important antioxidant role in cells [24]. The regulation of plant responses to stress varies considerably between species and involves complex coordination processes. In particular, the key pathways and genes underlying the differences in salt tolerance between *G. inflata* and *G. uralensis* are poorly understood, and transcriptome sequencing can be used to systematically elucidate the mechanisms underlying these differences.

In a soil environment, roots are the initial part of a plant that responds to stress and play a crucial role in stress response [25]. In rice, suberization is enhanced under salt stress conditions, and the extent of suberin deposition in primary roots is negatively correlated with Na^+^ uptake into shoots, which can potentially alleviate stress damage to the aboveground plant parts [26]. Transcriptome sequencing is a practical method for identifying potential functional genes in living organisms, and these genes can be selected by genetic engineering to cultivate salt-resistant plants [27]. In transcriptome analysis, a combination of biological analysis methods can identify target genes from transcriptome data. Weighted gene co-expression network analysis (WGCNA) can associate gene networks with stress traits to identify genes that exhibit core synergistic changes [28]. Plants utilize different regulatory mechanisms in response to salt stress at different developmental stages [29]. For analyzing trends, the STEM tool can more accurately determine the dynamic changes in gene expression, but the analysis of all genes is difficult because there are too many trends. The Mfuzz tool enables fuzzy clustering of all genes, but this approach is not sufficiently precise. Therefore, the combination of STEM and Mfuzz allows a better spatiotemporal transcriptome analysis. Based on transcriptome data, a combination of the WGCNA, STEM, and Mfuzz analysis methods could be used to effectively screen the differentially expressed genes (DEGs) and pathways between two species of licorice under salt stress.

In this study, *G. uralensis* and *G. inflata* (which exhibit higher salt tolerance than other licorice species), were used as research materials. The present study included analyses of the traits and related indicators (dry weight, malondialdehyde level, ion content, total flavonoids content) of the two licorice species at different developmental stages in combination with an analysis of the root transcriptome; additionally, comprehensive analyses were performed via WGCNA combined with the Mfuzz method, and the results were cross-validated with the results from a STEM analysis. Through these methods, the key genes in the pathway of interest were screened. This research is expected to provide guidance for the cultivation of high-quality salt-tolerant licorice and novel insights for saline-alkaline land improvement.

## Results

### Differences in the dry weight and MDA content between *G. inflata* and *G. uralensis*

**Fig. 1:**
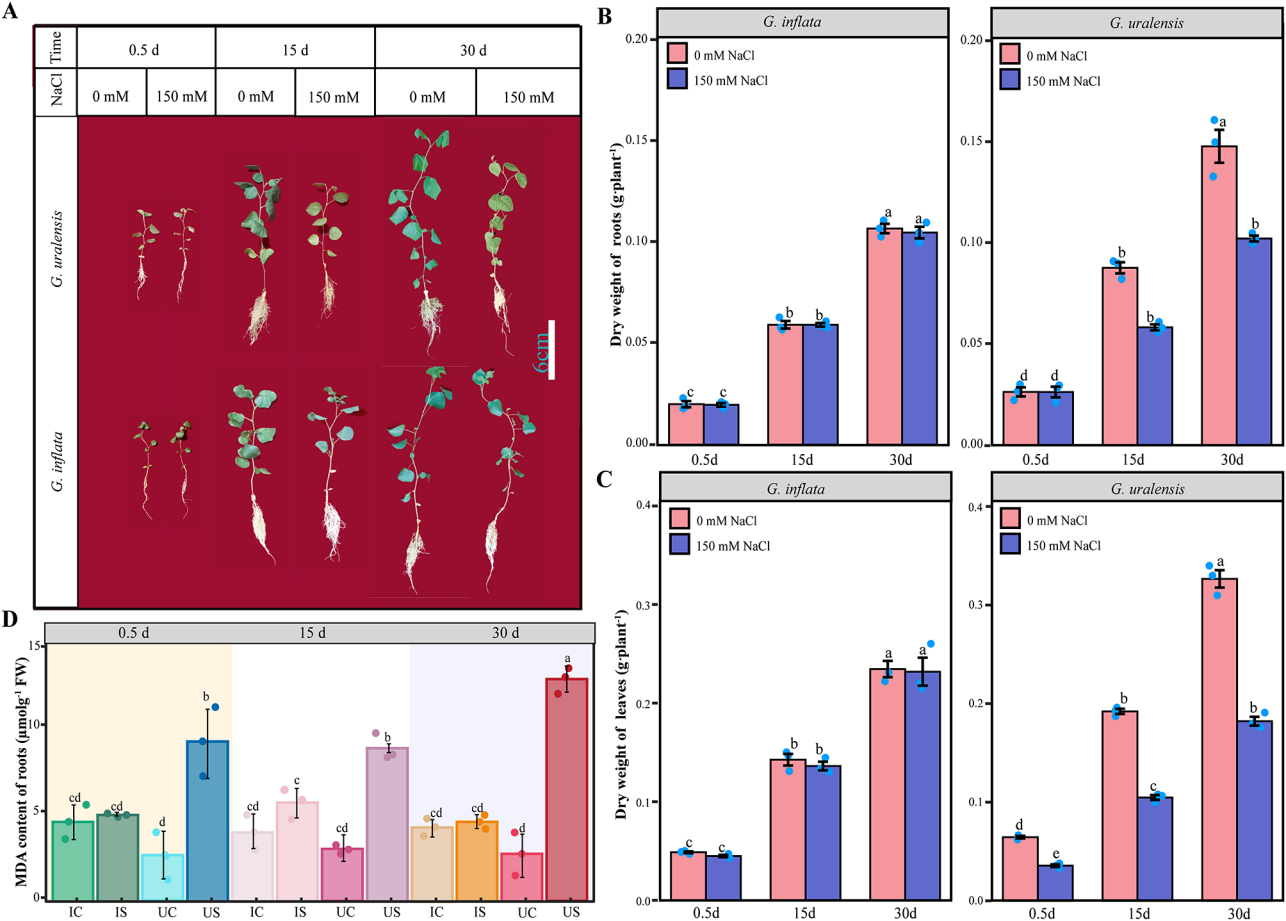
Morphological observations, dry weight and MDA content of *G. inflata* and *G. uralensis* after 0.5 d, 15 d, and 30 d of salt stress. **A** Morphological observations. **B** Dry weight of the roots. **C** Dry weight of the leaves. **D** MDA content of *G. inflata* (I) and *G. uralensis* (U) under salt treatment (S) and the control conditions (C). *Note*: In **B**-**D**, the Tukey test was performed by using the R-function Tukey HSD. Columns marked with the same letters were not significantly different based on the Tukey test (*p* < 0.05). The values are the means ± SEs (*n* = 3)

The morphological traits, dry weight, and malondialdehyde (MDA) content of *G. uralensis* and *G. inflata* were assessed after 0.5 d, 15 d, and 30 d of growth under various conditions. The 150 mM salt treatment for 15 d and 30 d resulted in decreases in the *G. uralensis* biomass compared with the 0 mM salt treatment; specifically, the root and leaf dry weights decreased by 34% and 46%, respectively, at 15 d and by 31% and 44%, respectively, at 30 d (Fig. 1A-C). Moreover, salt stress increased the MDA content in *G. uralensis* by 353%, 295%, and 456% at the three different time points (Fig. 1D). In contrast, salt treatment did not have a discernible effect on the morphological characteristics of *G. inflata*, and neither the dry weight nor the MDA content after salt exposure differed significantly from that of the control (Fig. 1A-D). These findings showed that the application of 150 mM NaCl had a small effect on the development of *G. inflata* but restricted the development of *G. uralensis* roots and leaves. Additionally, salt stress may have enhanced the lipid peroxidation levels in the roots of *G. uralensis*.

### Differences in the Na^+^, K^+^, Ca^2+^ and Mg^2+^ levels between *G. inflata* and *G. uralensis*

**Fig. 2:**
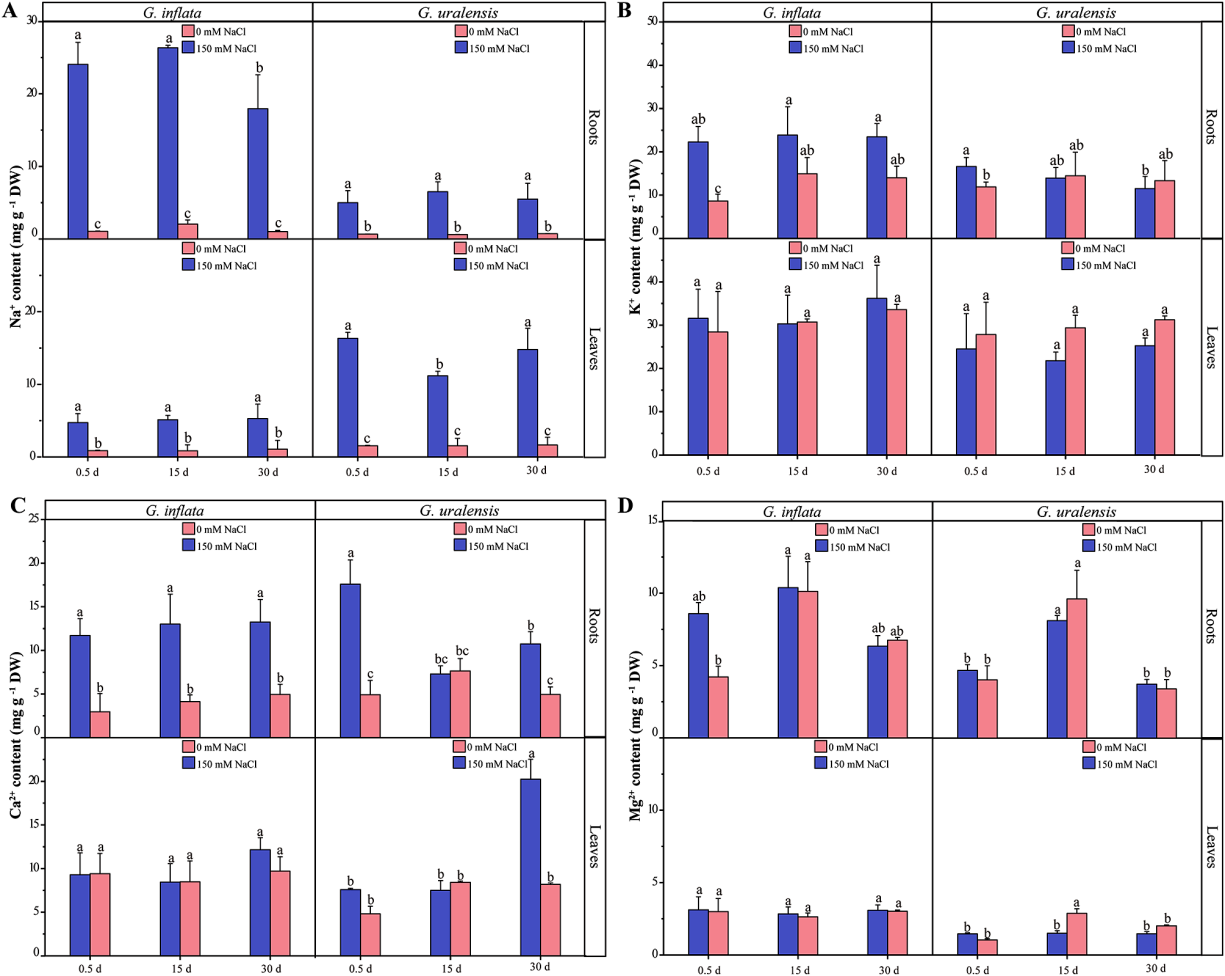
Ionic content (Na^+^, K^+^, Ca^2+^, Mg^2+^) in the salt treatment and control groups of *G. inflata* and *G. uralensis*. **A** Na^+^. **B** K^+^. **C** Ca^2+^. **D** Mg^2+^. *Note*: the Tukey test was performed by using the R-function Tukey HSD. Columns marked with the same letters were not significantly different based on the Tukey test (*p* < 0.05). The values are the means ± SEs (*n* = 3)

The Na^+^ concentration in the roots and leaves of both licorice species after exposure to salt was greater than that those under control conditions (Fig. 2A). However, the Na^+^ concentration in the *G. inflata* roots was 4.80, 5.14, and 3.28 times greater than that in the leaves under the same treatment at 0.5 d, 15 d, and 30 d, respectively, whereas the Na^+^ content in the *G. uralensis* roots was only 30.6%, 58.3%, and 35.7% of that in the leaves at 0.5 d, 15 d, and 30 d, respectively (Fig. 2A). Taken together, these findings indicate that under high salt conditions, *G. inflata* accumulates Na^+^ mainly in the roots and can transfers less Na^+^ to the ground, which is the opposite of the trend observed for *G. uralensis*. At 15 d, the Ca^2+^ contents in *G. inflata* and *G. uralensis* under salt treatment were 296% and 97% greater than those under the control conditions, respectively (Fig. 2C). At 15 d and 30 d, the K^+^ concentration in *G. uralensis* did significantly differ between the treatments. The K^+^ and Ca^2+^ contents in the *G. inflata* roots after salt treatment were greater than those in the control roots, whereas the Mg^2+^ levels exhibited the same trend in both licorice species (Fig. 2B-D). These results showed that both licorice species maintained their aboveground K^+^ supply under 150 mM salt, whereas *G. inflata* exhibited a greater K^+^/Na^+^ ratio to maintain normal growth and development.

### Differences in the total flavonoids content between *G. inflata* and *G. uralensis*

**Fig. 3:**
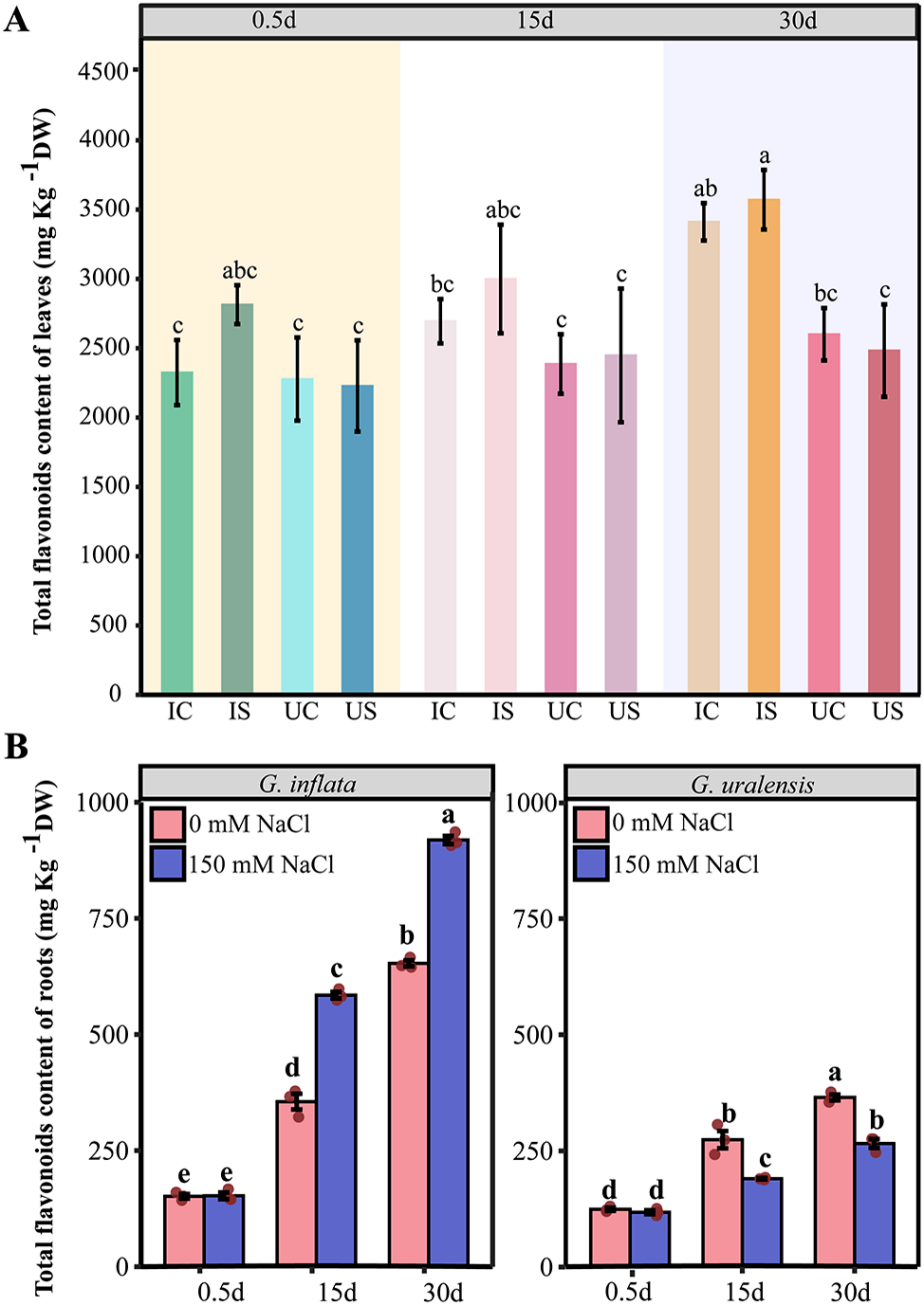
Total flavonoids contents of *G. inflata* and *G. uralensis* after 0.5 d, 15 d, and 30 d of salt stress. **A** Total flavonoids content of leaves in *G. inflata* (I) and *G. uralensis* (U) under salt treatment (S) and the control conditions (C). **B** Total flavonoids content of the roots. *Note*: the Tukey test was performed by using the R-function Tukey HSD. Columns marked with the same letters were not significantly different based on the Tukey test (*p* < 0.05). The values are the means ± SEs (*n* = 3)

The total flavonoids concentrations in the roots and leaves of *G. uralensis* after salt treatment for 30 d were markedly lower than the control concentrations; however, the concentrations in the roots of *G. inflata* were markedly greater than that in the control roots at 15 d and 30 d (1.407 and 1.645 times higher, respectively), and the total flavonoids concentration in the salt-treated *G. inflata* leaves at 30 d was 1.170 times greater than that in the control leaves (Fig. 3A, B). Unlike in *G. uralensis*, treatment with 150 mM NaCl promoted the accumulation of total flavonoids in *G. inflata* roots and leaves (Fig. 3A, B).

### Comparison of the transcriptomes and identification of DEGs

The root transcriptomes of the two licorice species were sequenced using 12 × 3 (3 biological replicates) libraries. The number of raw reads ranged from 23,003,802 to 33,581,031, whereas the number of clean reads ranged from 22,485,995 to 33,097,419, with a Q30% greater than 92.11%. The percentage of GCs ranged from 44.97 to 45.89%, indicating that the sequencing data were of high quality (Supplementary Table S1). Moreover, 85.02–89.2% of the high-quality read fragments were mapped to the *G. uralensis* reference genome (Supplementary Table S2).

**Fig. 4:**
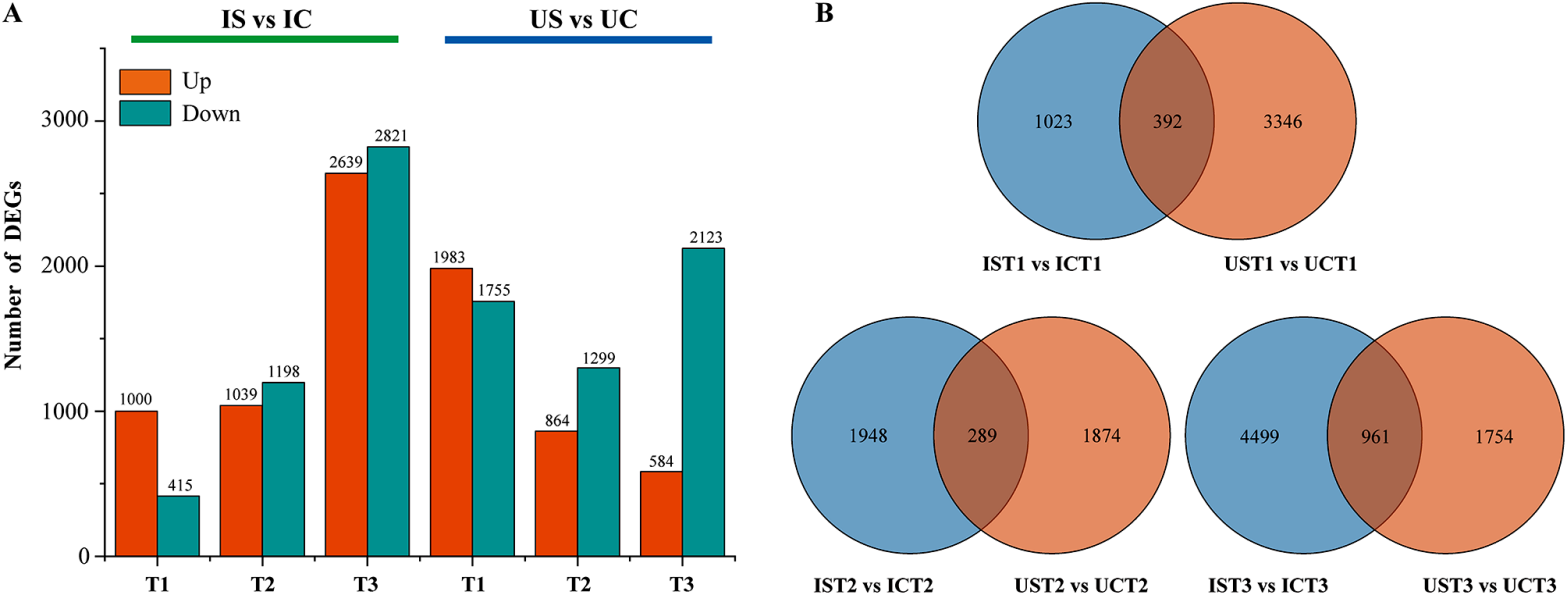
Statistics of DEGs of *G. inflata* (I) and *G. uralensis* (U) under salt stress (**A**) and Venn analysis of salt response-related DEGs of *G. inflata* and *G. uralensis* after exposure to salt stress for the same duration (**B**)

In total, 16,086 salt responsive DEGs were identified in both licorice species using the parameter FPKM values to measure the gene expression levels. As the duration of salt stress increased, the number of genes whose expression levels substantially changed in *G. inflata* increased, whereas the number of genes whose expression levels markedly changed in *G. uralensis* decreased (Fig. 4A). According to the Venn diagram, 392 DEGs were shared by both licorice species after 0.5 d of salt stress, and 1023 and 3346 DEGs were unique to *G. inflata* and *G. uralensis*, respectively. After 15 d of salt exposure, 289 DEGs were shared by both licorice species, 1984 DEGs were unique to *G. inflata*, and 1874 DEGs were unique to *G. uralensis*. After 30 d of salt treatment, a total of 961 DEGs were shared between the two licorice species, and 4499 DEGs and 1745 DEGs were unique to *G. inflata* and *G. uralensis*, respectively (Fig. 4B).

### Mfuzz and WGCNA conjoint analysis

**Fig. 5:**
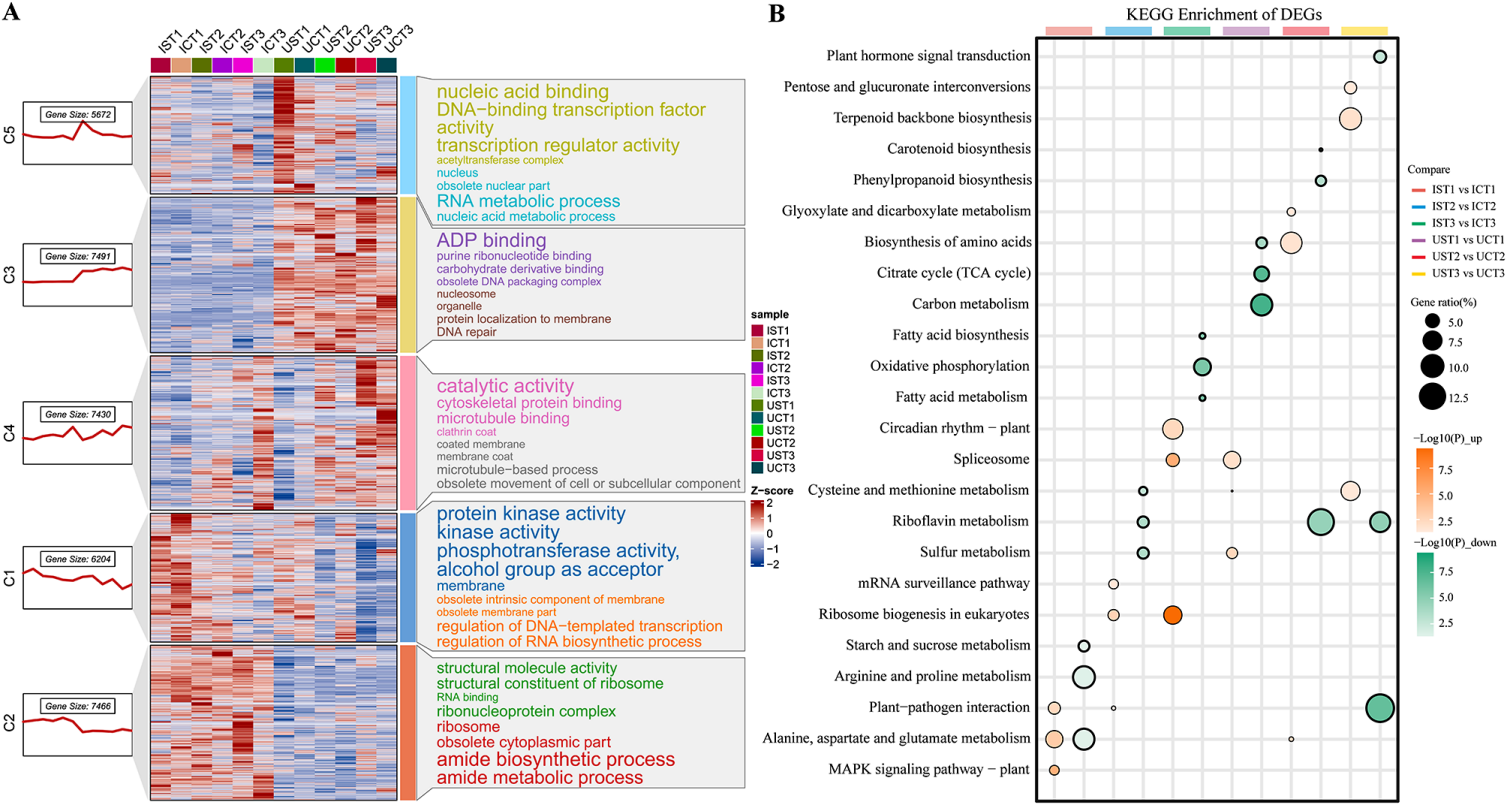
Functional analysis of the DEGs following Mfuzz analysis. **A** Gene clusters C1-C5 identified through Mfuzz analysis. The top eight Gene Ontology (GO) terms for each cluster were annotated based on a significance level of *p* < 0.05. According to these annotations, the font size of each gene cluster is directly proportional to the inverse of its p-value, which means that a larger font size indicates a smaller p-value. **B** Bubble plots of the top 3 KEGG pathway enrichment (orange is up-regulated, green is down-regulated) for the different comparison groups based on Venn analysis

Mfuzz clustering and GO analysis of the DEGs, yielded a set of genes with different expression trends and annotation information (Fig. 5A). Positive z-scores are typically employed to indicate potential upregulation or increased activity of gene expression under specific conditions. Across both C1 and C2, irrespective of whether the plants were exposed to 0 mM or 150 mM NaCl, the majority of the DEGs exhibited upregulated expression in *G. inflata* but downregulated expression, in *G. uralensis*. Furthermore, many DEGs were identified. In *G. inflata*, the expression of these genes in C1 and C2 was greater in the salt-treated group (IS) than in the control group (IC) (Fig. 5A). C1 and C2 were more likely to be our target gene sets (Fig. 5A). Annotation of the up-and down-regulated genes in the different comparison groups by KEGG, the “MAPK signing pathway” and “circadian rhythm” were markedly up-regulated in *G. inflata* and that the “TCA cycle”, “carbon metabolism” and “carotenoid biosynthesis” were down-regulated in *G. uralensis* (Fig. 5B).

**Fig. 6:**
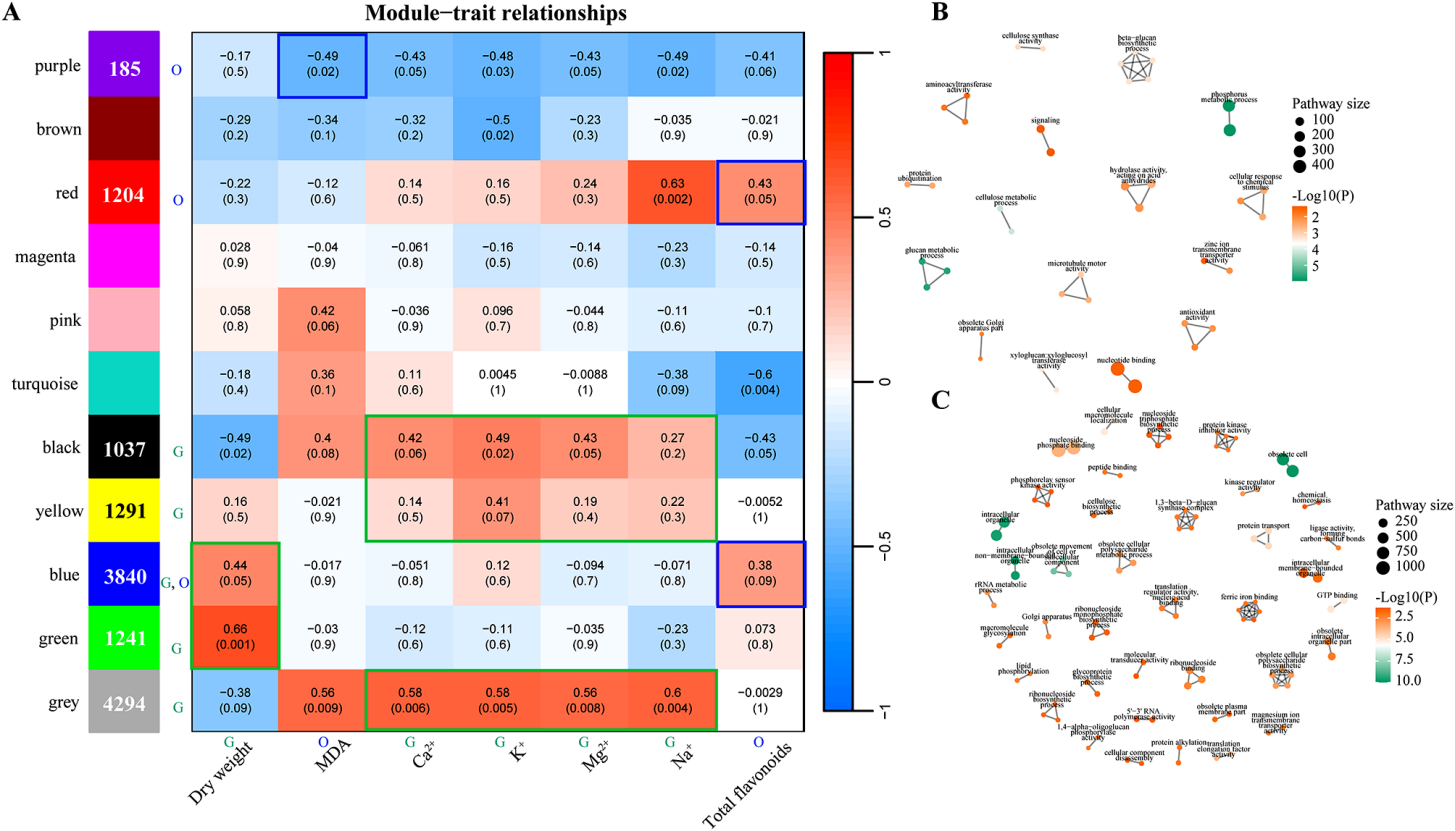
WGCNA analysis and GO network cluster analysis. **A** Relationship of co-expression modules to trait metrics in the WGCNA analysis. The number in the box represents the correlation between the module and the trait, and the number in the bracket represents the p-value for that correlation. Red indicates a positive correlation, blue indicates a negative correlation. In the figure, the letter “G” indicates the “growth” group and “O” indicates the “oxidation” group. **B** aPEAR analysis (GO enrichment) of the DEGs in the “growth” group. **C** aPEAR analysis (GO enrichment) of the DEGs in the “oxidation” group. *Note*: In **B** and **C**, the nodes represent the significant pathways, and the edges represent the similarities between pathways

For an analysis at a finer resolution, we performed a WGCNA of the DEGs. A total of 11 gene-phenotype co-expression modules were generated, and these modules were categorized into “growth” and “oxidation” groups. Based on the characteristics of the traits, we screened the gene sets most likely associated with high salt tolerance in *G. inflata* from the various modules. Subsequently, we determined the dry weight, MDA content, ion content, and total flavonoids. Among these parameters, dry weight and ion content were found to be closely linked to growth and development, and we marked these parameters as “G”. The modules significantly positively correlated with them were limited to blue, green, grey, yellow, and black, which were collectively designated as the “growth” group (Fig. 6A). Additionally, MDA and total flavonoid are closely related to the antioxidant system, they were marked with an “O”. The only module showing a significant negative correlation with MDA was purple, while those significantly positively correlated with total flavonoid were red and blue, merged to form the “oxidation” group (Fig. 6A). The GO interconnected cluster networks of the two groups were generated after aPEAR analysis of the gene sets of the two groups (Fig. 6B, C). Overall, the “oxidation” group had more similar GO clusters, mainly “ferric iron binding”, “obsolete cellular polysaccharide biosynthetic process” and “1,3- beta-D-glucan synthase complex”, while the highest similarity was found in the “growth” group for the “beta-glucan biosynthetic process” pathway (Fig. 6B, C). This suggests that these GO annotations and their corresponding gene sets contribute more to the enrichment of the two groups.

**Fig. 7:**
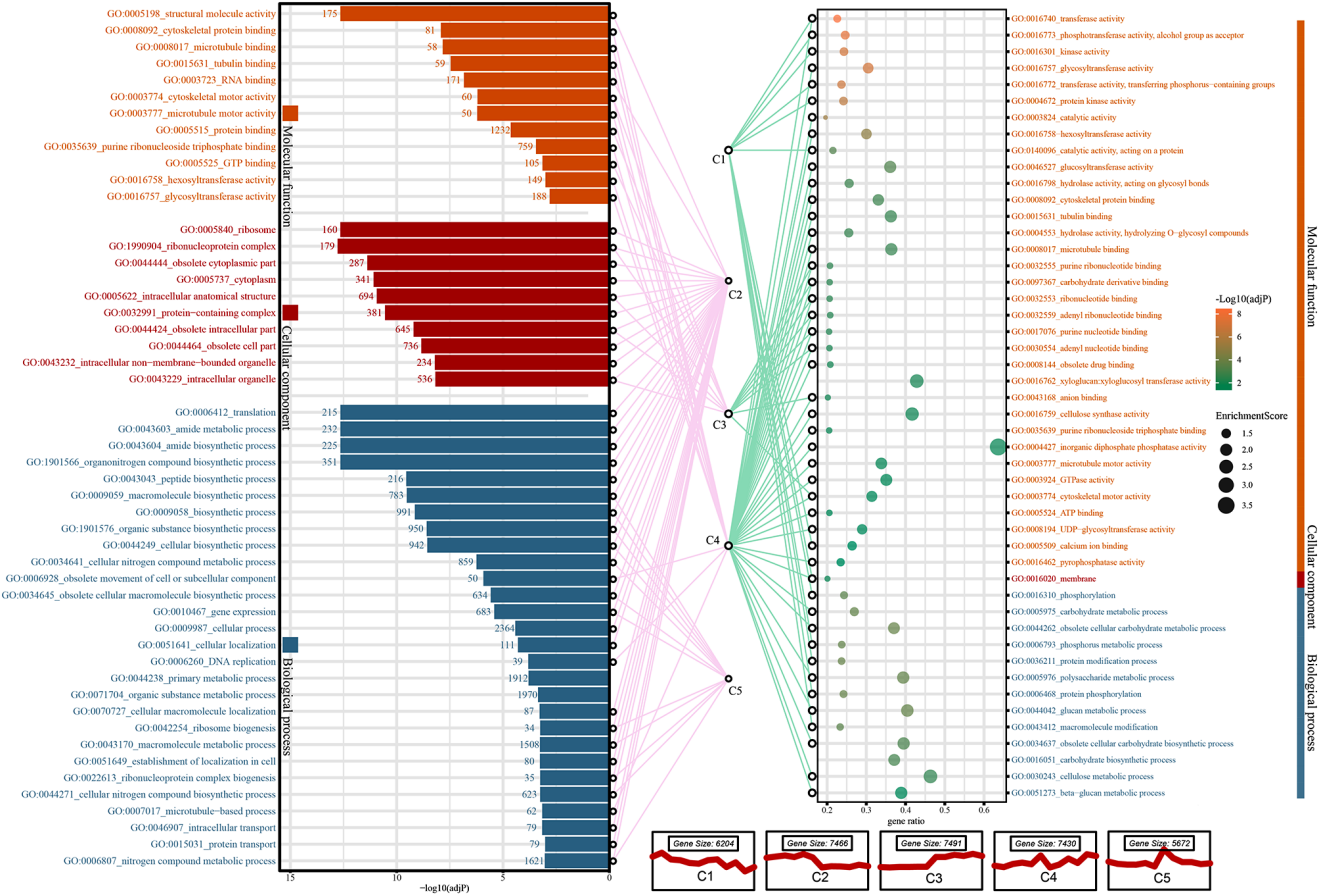
Significantly enriched GO terms (adjusted p-value < 0.05) in the ‘oxidation’ group (left) and the “growth” group (right). *Note*: The connecting lines represent that the GO terms identified in the “oxidation” and “growth” groups are also found to be significantly enriched in the different trend gene sets (C1-C5) showing different trends identified by Mfuzz analysis

After the GO enrichment analysis of the “growth” and “oxidation” groups, all the GO annotations (adjP < 0.05) were analyzed; the MF term “structural molecule activity”, the BP term “ribosome”, and the CC term “translation” were the most significant terms obtained for the “oxidation” group, and the MF term “transferase”, the BP term “membrane”, and the CC item “phosphorylation” were the most significant terms found for the “growth” group (Fig. 7). Notably, the results from combined WGCNA and Mfuzz result (the same annotation was paired with a network connection), C1 was only co-matched with “growth” and C2 was only co-matched with “oxidation” (Fig. 7). This coincidence is intriguing. C1 and C2 represent gene sets that are highly expressed in *G. inflata* but expressed at low levels in *G. uralensis*. The enrichment analysis results aligned with the enriched gene sets from two merged traits in the WGCNA. This finding suggests that the shared enriched pathways resulting from the combination of Mfuzz and WGCNA may exhibit two distinct characteristics. First, these pathways showed higher gene expression levels in *G. inflata* than in *G. uralensis* and there may be rich response pathways in *G. inflata* under salt stress due to some higher expression of genes than control conditions. Second, there were two types of functions in which these highly expressed genes were significantly enriched, which are potentially related to growth and antioxidation (Fig. 7).

**Fig. 8:**
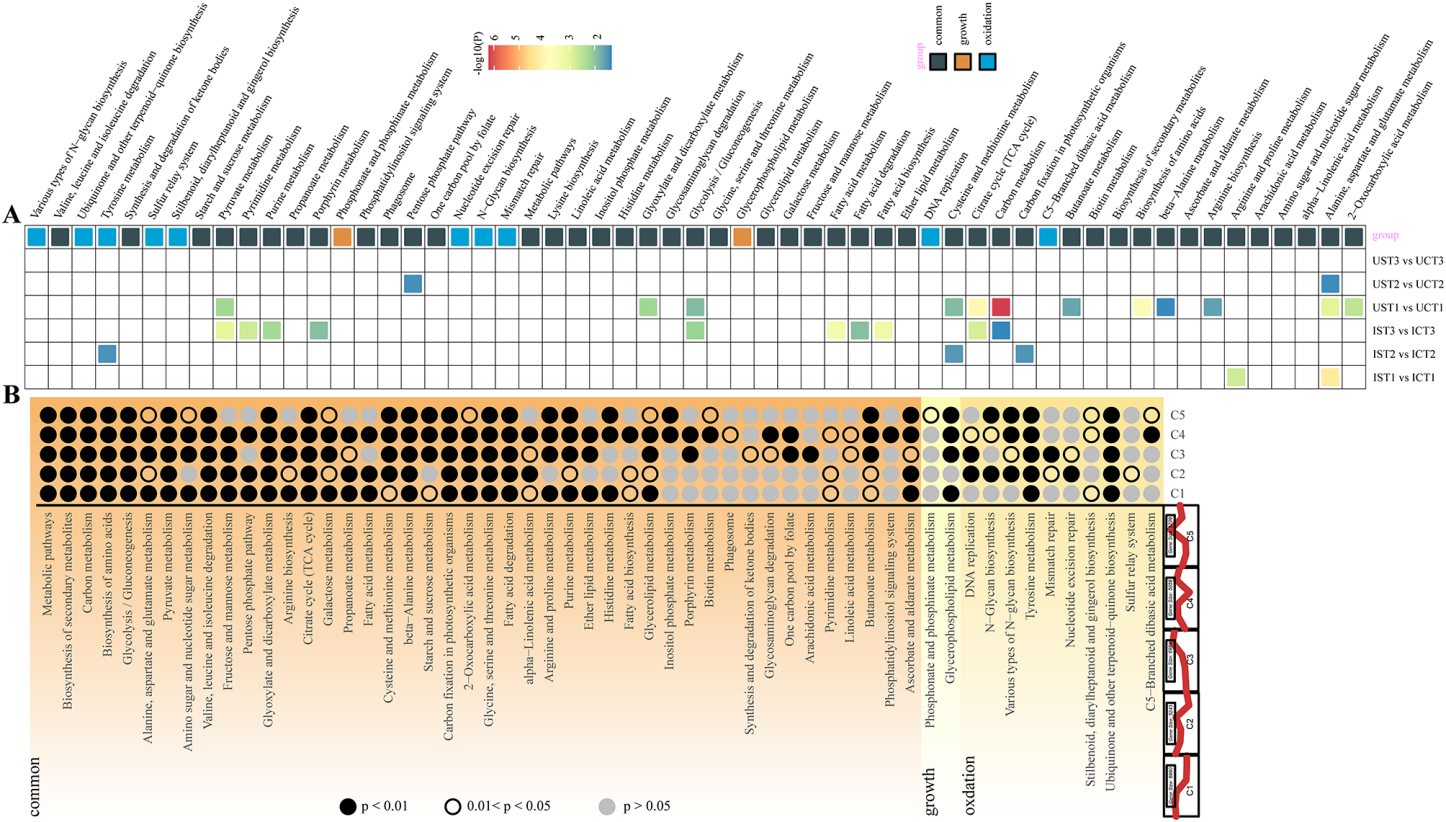
KEGG enrichment analysis of gene sets from the “oxidation” group and “growth” group by WGCNA. **A** All significant enrichment pathways (*p* < 0.05) from KEGG analysis of the “growth” group and “oxidation” group gene clusters are enriched and distributed in different comparison terms by Venn analysis. **B** All significant enrichment pathways (*p* < 0.05) from KEGG analysis of the “growth” group and “oxidation” group gene clusters are enriched and distributed in different comparison terms by Mfuzz analysis

All the annotations with *p* < 0.05 were categorized as “growth”, “oxidation”, and “common” (common annotations to both groups) term after the KEGG analysis of the “growth” and “oxidation” gene sets, respectively, and the enrichment of these pathways was viewed in the comparison samples (Fig. 8A). Notably, the enrichment situation of three moments in *G. uralensis* had the opposite trend of those in *G. inflata*: markedly enriched pathways were abundant in *G. uralensis* at T1, and 0 at T3, whereas they were more abundant in *G. inflata* at T3 and were present in small counts at T1 and T2 (Fig. 8A). In addition, “arginine and proline metabolism” at T1; “carbon fixation in photosynthetic organisms”, “tyrosineyrosine metabolism”, “cysteine and methionine metabolism” at T2; “glycolysis/ gluconeogenesis, “fatty acid metabolism”, “TCA cycle”, “carbon metabolism”, and various metabolic pathways at T3 were enriched only in *G. inflata* (Fig. 8A). We continued to look at the enrichment of these pathways come from WGCNA in Mfuzz’s 5 trends and found KEGG annotations specific to each of the 5 trends. Compared with C2, the C1 trend of “alanine, aspartate and glutamate metabolism”, “arginine biosynthesis”, “galactose metabolism”, and “starch and sucrose metabolism " was enriched (Fig. 8B). These organic solutes analyzed from the “growth” group, such as soluble sugars, amino acids and arginine may be also involved in the regulation of osmotic homeostasis under *G. inflata* salt stress.

In summary, the reason for the higher salt tolerance in *G. inflata* than in *G. uralensis* is more likely to be attributed to the responses of growth (carbon metabolism), osmoregulation (inorganic ion transport, organic solute metabolism), antioxidant (carotenoid synthesis, flavonoid synthesis) associated pathways and their DEGs.

### STEM analysis

Further categorization of the dynamic transcriptional profiles of the DEGs at the three-time points showed that 7772 DEGs of *G. inflata* were into three profiles: profile 1, which consisted of the DEGs that were consistently downregulated; profile 4, which consisted of the DEGs that were upregulated at T1 and T2; and profile 3, which consisted of the DEGs that were upregulated at T1 and T3 and downregulated at T2. The 7,543 DEGs of *G. uralensis* were markedly clustered into profile 1 (consistently downregulated) (Supplementary Fig. S1). The number of genes that were consistently downregulated were more in the roots of *G. uralensis* than in those of *G. inflata*, whereas the number of genes that were consistently upregulated were more in the roots of *G. inflata* than in those of *G. uralensis* (Supplementary Fig. S1). Therefore, we selected at least the abovementioned three profiles (1, 3, and 4) for further enrichment analyses.

A comparison of the GO enrichment data (profiles 1, 3, and 4) revealed that the salt response patterns of both licorice species were different (Supplementary Fig. S1B). A KEGG analysis was performed for both *G. inflata* and *G. uralensis*; a total of 5 major classes, 18 subclasses, and 118 pathways were annotated, and 35 pathways were significantly enriched (Supplementary Fig. S2). Pathways related to carbon fixation in photosynthetic organisms, the TCA cycle, pentose phosphate pathway (PPP), cellular organ-solute biosynthesis (isoline alkaloid biosynthesis, arginine and proline metabolism), nonenzymatic antioxidant anabolism (carotenoid biosynthesis, flavonoid biosynthesis), phytohormone signaling, and endothelial barrier formation (phenylpropane biosynthesis, fatty acid degradation) were enriched in *G. inflata* and *G. uralensis* (Supplementary Fig. S2B). None of these pathways showed the same trends in both licorice species, and the differential enrichment of these pathways may be critical for the differences in the salt responses of the two licorice species.

After mutual verification of the critical pathways identified via STEM analysis and the results obtained via Mfuzz and WGCNA conjoint analysis, the critical pathways identified via two biological analyses were highly compatible. These analyses scientifically demonstrated that, unlike *G. uralensis*, *G. inflata* has high resistance to salt stress through carbon metabolism (TCA cycle, pentose phosphate pathway, carbon fixation in photosynthetic organisms), endothelial barrier formation (phenylpropane biosynthesis, fatty acid metabolism), osmoregulation (isoline alkaloid biosynthesis, arginine and proline metabolism, transport of inorganic ions), non-enzymatic antioxidant synthesis (carotenoid biosynthesis, flavonoid biosynthesis), hormone signaling pathways.

### Identification of key pathways and genes

#### Key DEGs involved in carbon metabolism

**Fig. 9:**
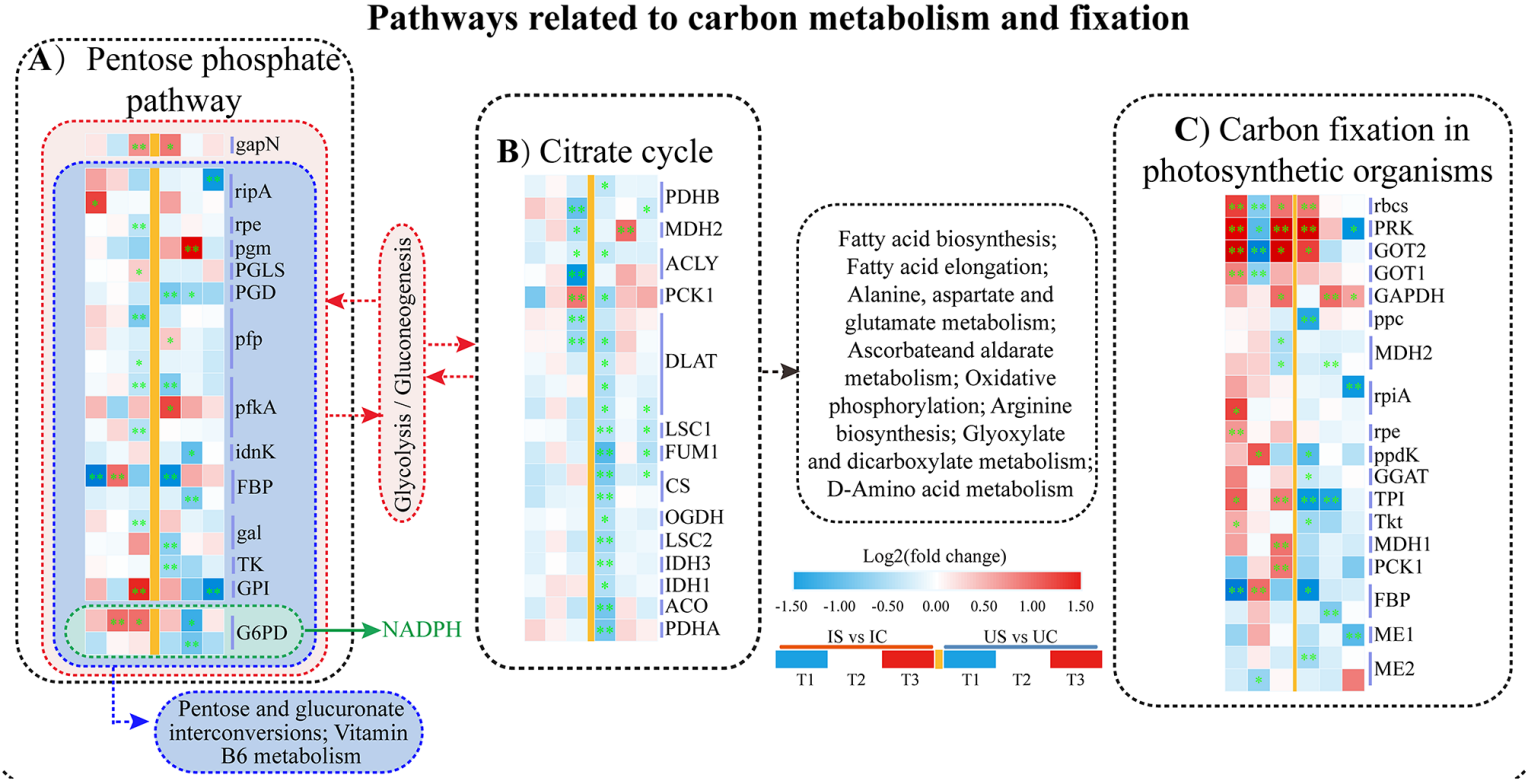
DEGs involved in carbon metabolism between *G. inflata* (I) and *G. uralensis* (U). **A** Pentose phosphate pathway. **B** TCA cycle. **C** Carbon fixation in photosynthetic organisms. *Note* Significant differences are shown by “*” (*p* < 0.05); highly significant differences are shown by “**” (*p* < 0.01)

PPP was identified with a total of 22 DEGs (Fig. 9A). We focused primarily on genes exhibiting significant differences (*p* < 0.05). At T1, T2, and T3, the expression of 1, 2, and 4 genes was increased in *G. inflata* roots, whereas that of 1, 0, and 6 genes was decreased. However, the expression of 3, 1, and 0 genes was increased and that of 5, 5, and 2 genes was decreased at T1, T2, and T3, respectively, in *G. uralensis* (Fig. 9A). Among these genes, the expression of the gene encoding the first key enzyme of this process, G6PD, was increased in *G. inflata* and decreased in *G. uralensis*. Twenty-one DEGs involved in the TCA cycle were significantly expressed, and only six DEGs in the TCA cycle showed reduced expression only at T3 in *G. inflata*, and 17, 0, and 5 genes showed reduced expression at T1, T2, and T3, respectively, in *G. uralensis* (Fig. 9B). The first rate-limiting enzyme of the cycle, citrate synthase, is encoded by citrate synthase gene, and the expression of this gene was downregulated in *G. uralensis*. Among the 22 DEGs associated with carbon fixation in photosynthetic organisms, 8, 2, and 7 DEGs were upregulated and 1, 5, and 2 DEGs were downregulated at T1, T2, and T3, respectively, in *G. inflata* roots, whereas 3, 1, and 1 DEGs were upregulated and 7, 3, and 3 DEGs were downregulated, respectively, in *G. uralensis* roots (Fig. 9C). Unlike the trend found in *G. inflata*, 150 mM salt treatment may largely inhibit the carbon metabolism (energy metabolism) processes in *G. uralensis* roots.

#### Key DEGs involved in Na^+^, K^+^, Ca^2+^ and Mg^2+^ transport

**Fig. 10:**
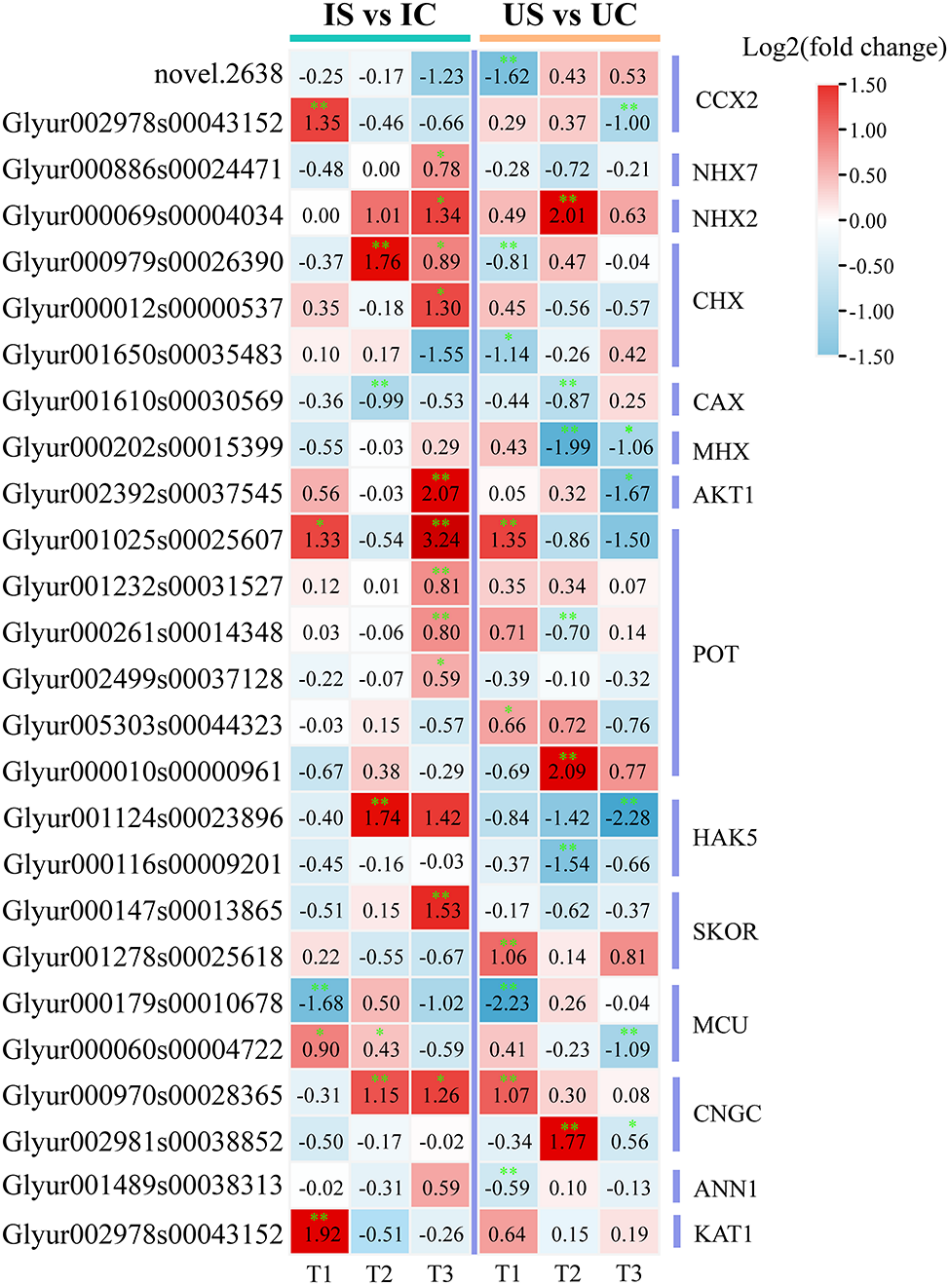
DEGs encoding ion transporters (Na^+^, K^+^, Ca^2+^ and Mg^2+^) of *G. inflata* (I) and *G. uralensis* (U) under salt stress. *Note*: Significant differences are shown by “*” (*p* < 0.05); highly significant differences are shown by “**” (*p* < 0.01). The number represents the log2(fold change) value of the gene differential expression multiple

In the roots of both licorice species, the adjustment of Na^+^, K^+^, and Ca^2+^ absorption or transport under salt exposure was found to involve a total of 26 DEGs (Fig. 10). The expression of NHX7, which regulates the extracellular efflux of Na^+^, and NHX2, which regulates the vesicular compartment of Na^+^, was increased in both licorice species, and more pronounced at T3 in *G. inflata* (Fig. 10). The expression of the cation/H (+) antiporter (CHX) genes, potassium channel (AKT1, KAT1) gene, and potassium transporter (POT, HAK5) genes were all increased in *G. inflata* and decreased in *G. uralensis* roots (Fig. 10). The expression of the cation/calcium exchanger 2 (CCX2), calcium uniporter protein 6 (MCU), cyclic nucleotide-gated ion channel (CNGC), annexin D1 (ANN1), and cation/proton exchanger (CAX) genes was decreased in *G. uralensis* but increased in *G. inflata*, except for CNGC, which showed significant upregulation in both licorice species (Fig. 10). This finding indicates that the roots of the two licorice species may show distinct ion uptake and transport mechanisms under salt stress, and unlike *G. uralensis*, 150 mM salt treatment of *G. inflata* led to upregulation of genes associated with K^+^ and Ca^2+^ transport and Na^+^ compartmentalization and cytoplasmic efflux.

Key DEGs involved in endothelial barrier formation.

**Fig. 11:**
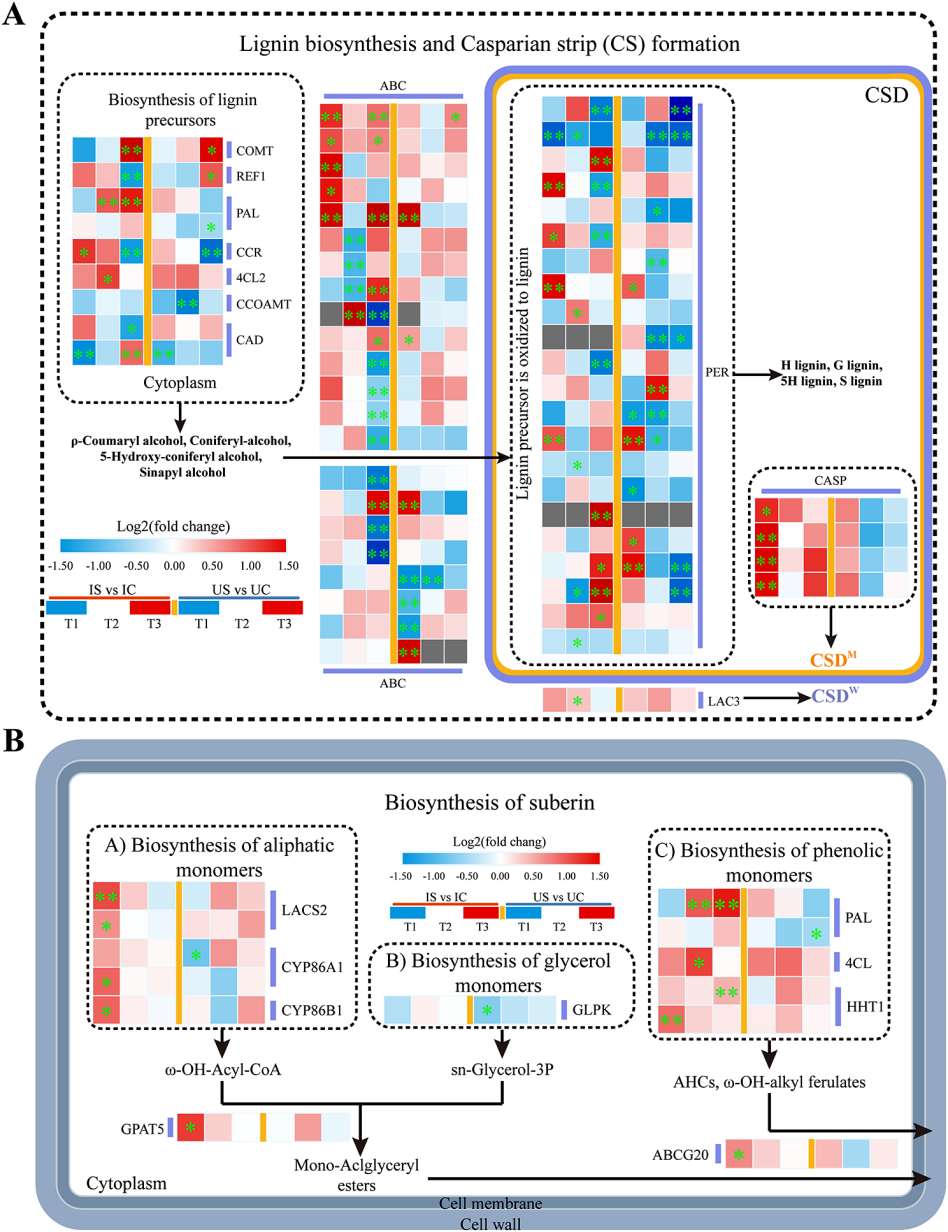
DEGs involved in endothelial barrier formation of *G. inflata* (I) and *G. uralensis* (U). **A** DEGs participating in Casparian strip formation. **B** DEGs participating in suberin lamellae formation. *Note*: Significant differences are shown by “*” (*p* < 0.05); highly significant differences are shown by “**” (*p* < 0.01)

The Casparian strip (CS) consists mainly of lignin polymers, whereas the suberin lamellae form from suberin polymers deposited inside cell walls [30, 31] and are associated with phenylpropane biosynthesis and fatty acid degradation pathways. A total of 58 DEGs were discovered in this study as the synthesis, transport, and oxidation of lignin precursors and participating in the development of the CS (Fig. 11A). Under salt treatment, nine genes were differentially expressed in the two licorice species especially PAL and 4CL (Fig. 11A). Among the ABC transporters, the numbers of the DEGs with significantly upregulated expression at T1, T2, and T3 were 5, 1, and 6 in *G. inflata* and 3, 0, and 1 in *G. uralensis* roots, respectively. Peroxidase (PER) genes are involved in regulating the oxidation of lignin precursors to lignin, and at T1, T2, and T3, the numbers of genes that were markedly up- and downregulated were 4, 1, and 5 and 1, 4, and 4, respectively, in *G. inflata* roots but 4, 1, and 0 and 2, 6, and 5, respectively, in *G. uralensis* roots (Fig. 11A). The expression of all four CS domain protein (CASP) genes regulating lignin in the CS forming membrane domain (CSDM) was markedly increased at T1 in *G. inflata* roots, whereas no significant differences were observed in *G. uralensis* roots. In addition, laccase3 (LAC3) gene expression was increased at T2 in *G. inflata* roots, and the difference was not significant in *G. uralensis* roots. Compared with the results for *G. uralensis*, the results for *G. inflata* showed that 150 mmol/L salt treatment may have created more favourable conditions for the translocation and oxidation of lignin precursors in the roots and led to the formation of membrane domains to promote the development of CS.

Within fatty acid metabolism, twenty-six DEGs, including the cytochrome P450 86B1 (CYP86B1) gene and long-chain acyl-CoA synthetase 2 (LACS2) gene, were found; the products of these genes catalyse the biosynthesis of the aliphatic suberin monomers ω-hydroxy-fatty acids in the roots of both licorice species under salt treatment, and these genes were markedly upregulated in *G. inflata* only at T1, whereas CYP86A1 was markedly downregulated in *G. uralensis* (Fig. 11B). In addition, the DEGs involved in suberin phenolic monomer synthesis and extracellular transport of related substances in the roots of both licorice species showed significant upregulation in *G. inflata* (PAL;4CL; Omega-hydroxy palmitate O-feruloyl transferase, HHT1; ABCG20) and significant downregulation in *G. uralensis* (PAL) (Fig. 11B). The GPAT5 gene, which is related to the synthesis of a suberin base component, glycerol, in the roots of both licorice species, was markedly upregulated at T1 in *G. inflata*. The GLPK gene was markedly downregulated at T1 in *G. uralensis* (Fig. 11B). Unlike the trend in *G. uralensis*, 150 mM salt treatment created favourable conditions for the biosynthesis of suberin phenolic monomers and the aliphatic suberin monomers ω-hydroxy-fatty acids and glycerol and promoted the extracellular transport of these compounds at T1 in *G. inflata*, leading to the formation of suberin polymers.

#### Key DEGs involved in the metabolism of osmoregulatory substances

**Fig. 12:**
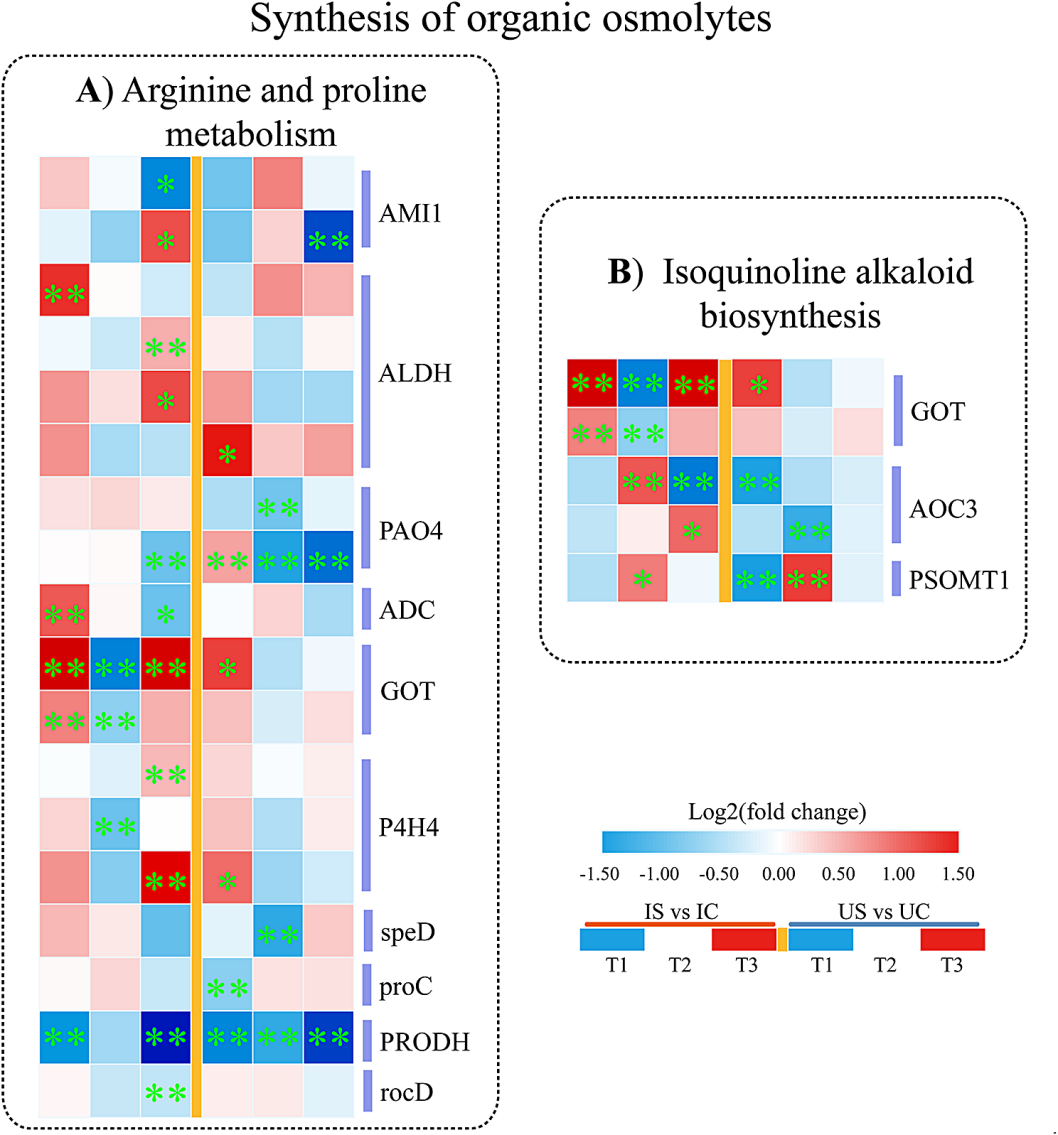
DEGs participating in organic osmolyte biosynthesis in *G. inflata* (I) and *G. uralensis* (U). **A** Arginine and proline metabolism. **B** Isoquinoline alkaloid biosynthesis. *Note*: Significant differences are shown by “*” (*p* < 0.05); highly significant differences are shown by “**” (*p* < 0.01)

Our analysis identified 21 DEGs associated with the metabolic processes of osmotic regulatory substances in both licorice species under salt exposure, and these processes included the metabolism of arginine and proline and the biosynthesis of polyamines and isoquinoline alkaloids (Fig. 12). The increase in the expression of the aspartate aminotransferase (GOT) gene, which participates in arginine synthesis, was more significant at T1 and T3 in *G. inflata* than in *G. uralensis*, whereas the proC (encoding P5C reductase) gene and ornithine aminotransferase (rocD) gene, which are related to proline biosynthesis in roots, were markedly downregulated at T1 and T3 in *G. uralensis* (Fig. 12A). In arginine degradation, the Amidase 1 (AMI1) gene exhibited opposite but significant differences in both licorice species at T3, and the related genes involved in proline depletion, the GOT and prolyl 4-hydroxylase 4 (P4H4) genes, were markedly downregulated at T2 only in *G. inflata*, whereas the proline dehydrogenase (PRODH) gene showed significant downregulation in the roots of both licorice species (Fig. 12A). In addition, the polyamine oxidase 4 (PAO4) gene, the arginine decarboxylase (ADC) gene, and the S-adenosylmethionine decarboxylase proenzyme (speD) gene, which are related to polyamine synthesis, were downregulated at T2 or T3 in both licorice species. The aldehyde dehydrogenase (ALDH) gene, the product of which synthesizes γ-aminobutyric acid, was significantly upregulated in *G. inflata* but not in *G. uralensis* at T3, whereas genes related to isoquinoline alkaloid synthesis (GOT, AOC3, and PSMOT1) showed more significant upregulation in *G. inflata* (Fig. 12B).

#### Key DEGs involved in the metabolism of non-enzymatic antioxidants

**Fig. 13:**
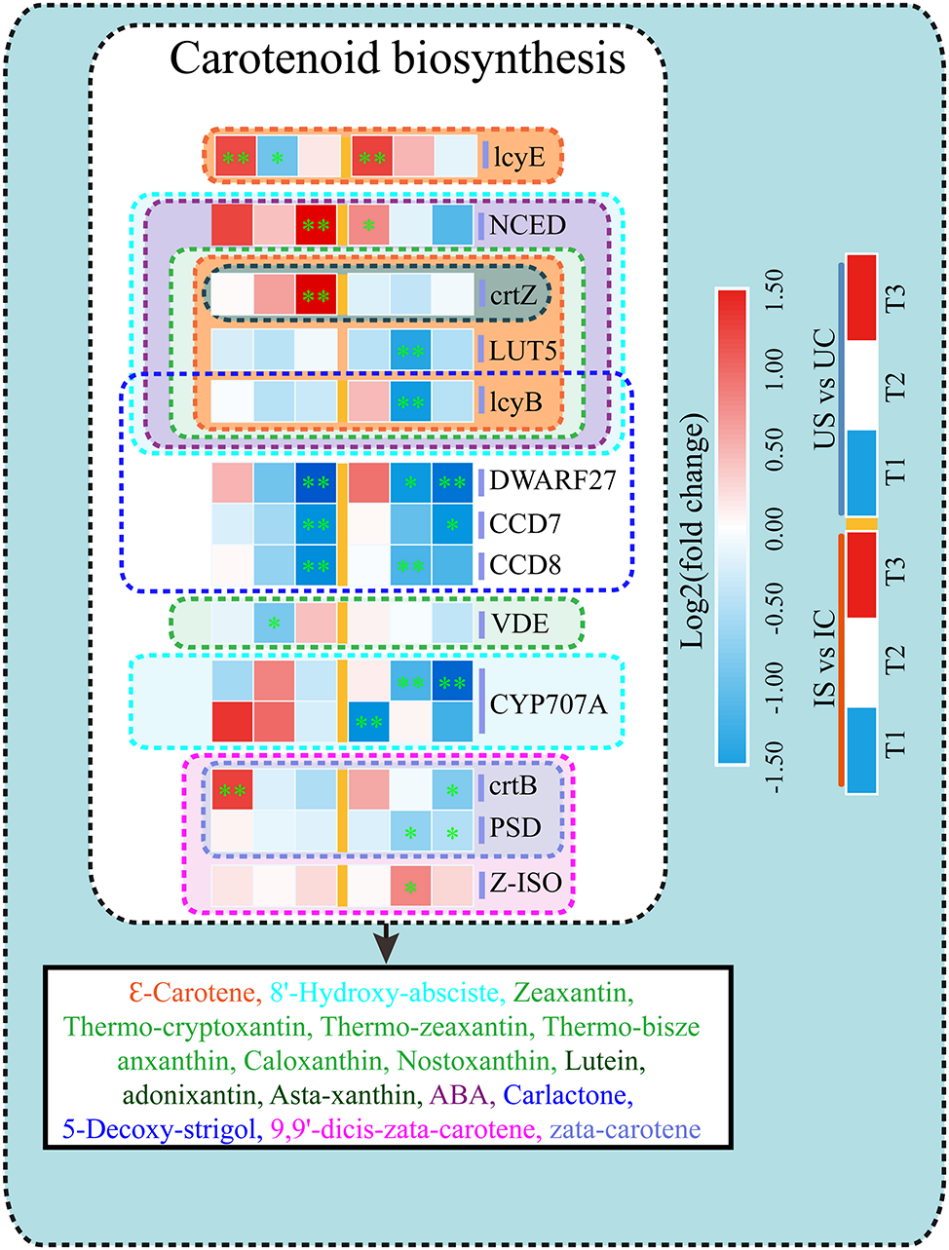
DEGs involved in carotenoid biosynthesis in *G. inflata* (I) and *G. uralensis* (U). *Note*: Significant differences are shown by “*” (*p* < 0.05); highly significant differences are shown by “**” (*p* < 0.01)

Fourteen DEGs associated with the metabolism of antioxidant carotenoids were identified in the roots of both licorice species under salt exposure (Fig. 13). The key genes of the lycopene synthesis pathway include the 15-cis-phytoene desaturase (PDS), 15-cis-phytoene synthase (crtB), and 15-cis-zeta-carotene isomerase (Z-ISO) genes; crtB and PDS were downregulated in *G. uralensis* only, but the expression of crtB was increased at T1 in *G. inflata*. The product of the lycopene epsilon cyclase (lcyE) gene synthesizes δ-carotene, and that of the lycopene beta cyclase (lcyB) gene synthesizes 7,8-dihydro-β-carotene; lcyB was more significantly downregulated only in *G. uralensis* at T2. The beta-carotene hydroxylase (crtZ) gene, the LUTEIN DEFICIENT 5 (LUT5) gene, and the violaxanthin de-epoxidase (VDE) gene are involved in lutein metabolism; crtZ was upregulated only in *G. uralensis* at T3, whereas LUT5 was downregulated only in *G. uralensis* at T2. Among the genes involved in ABA synthesis and degradation, the 9-cis-epoxycarotenoid dioxygenase NCED (NCED) gene was markedly upregulated in *G. inflata* at T3, whereas the CYP707A (abscisic acid 8’-hydroxylase) gene showed significant downregulation only in *G. uralensis* (Fig. 13).

#### Key DEGs involved in the biosynthesis of flavonoids

**Fig. 14:**
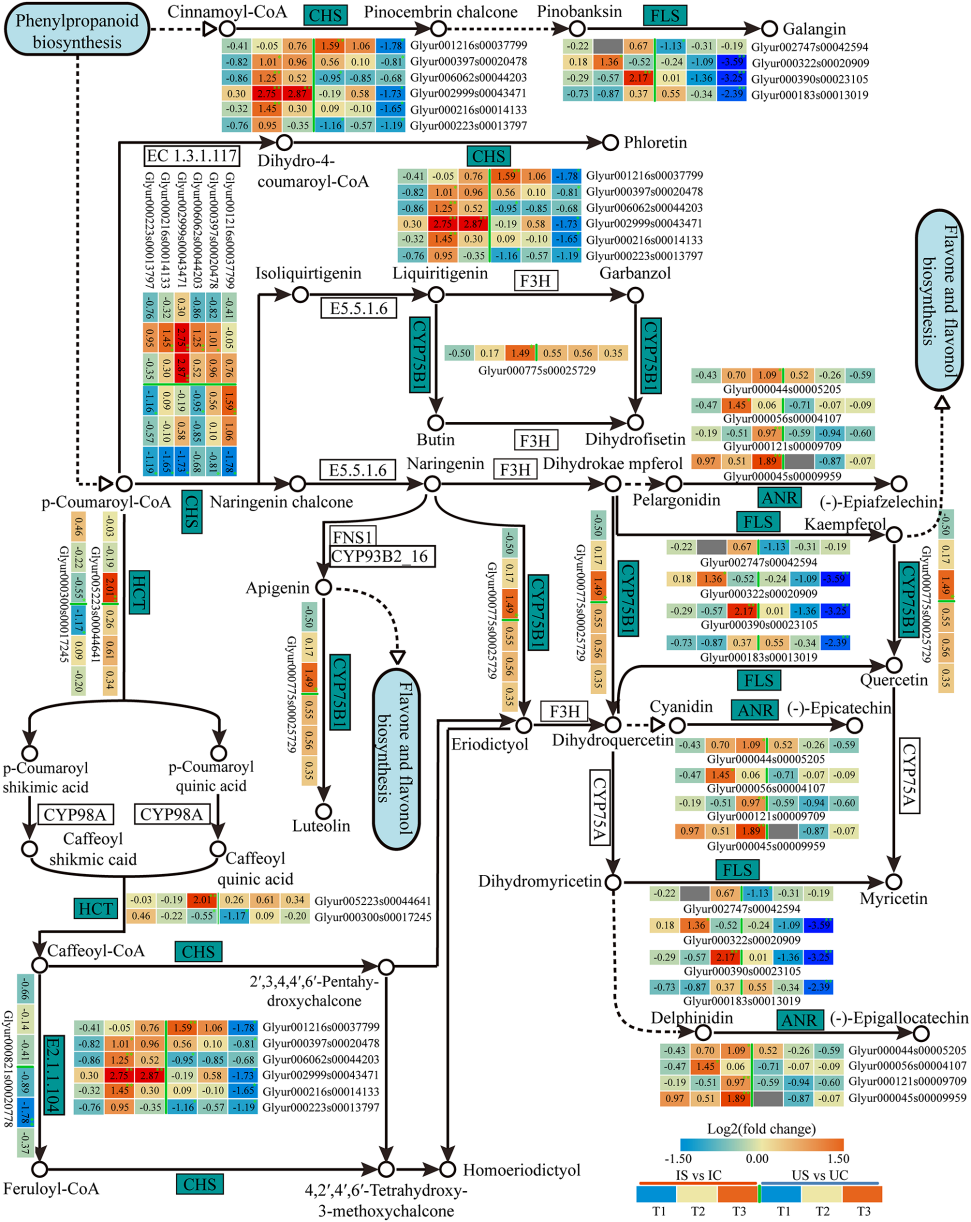
Heatmap of the DEGs related to the flavonoids biosynthesis pathway in *G. inflata* (I) and *G. uralensis* (U). *Note*: Significant differences are shown by “*” (*p* < 0.05); highly significant differences are shown by “**” (*p* < 0.01)

Among the genes involved in flavonoids biosynthesis, we identified 8 genes that showed differential expression in the roots of both licorice species under salt exposure (Fig. 14). Six CHS genes, the key genes of the pathway, were markedly downregulated in *G. uralensis* but significantly upregulated at T2 or T3 in *G. inflata*, and two HCT genes, which are related to acylation, were upregulated only at T3 in *G. inflata* (Fig. 14). Four FLS genes, four ANR genes, one CYP75B1 gene, and one CCoAOMT9 (EC2.1.1.104) gene involved in the synthesis of various flavonoids in plants showed upregulation in *G. inflata* and downregulation in *G. uralensis* (Fig. 14). In addition, two PAL genes and one 4CL gene, as key genes for the synthesis of flavonoid precursors in the phenylpropane biosynthesis pathway, also showed significant downregulation in *G. uralensis* roots only but significant upregulation at T2 or T3 in *G. inflata* roots (Supplementary Fig. S3). This finding indicates that the biosynthesis of flavonoids and their precursors were promoted in *G. inflata* roots and inhibited in *G. uralensis* roots under 150 mM salt treatment.

### Transcription factors

In total, 411 transcription factors (TFs) were differentially expressed in the roots of at least one of the two licorice species under salt exposure, and only 82 of these TFs were differentially expressed in both species. The main gene families responding to salt exposure, namely, the Myb_DNA-binding, AP2, bHLH, WRKY, and bZIP gene families, were similar at the three-time points (Supplementary Fig. S4). This finding indicates that the gene families regulating TFs in response to salt stress in the roots of both licorice species are similar, but the specific regulatory mechanisms are markedly different, and the number of TFs involved in the response to salt exposure was markedly higher at T3 in *G. inflata* roots than in *G. uralensis*. Further analysis of the dynamic expression trends of these TFs identified seven TFs that were enriched in *G. uralensis* profile 1 but not in *G. inflata* profile 1, which belonged to the FAR1 (1), HSF (5), and LSD (1) gene families; two TFs that were enriched in *G. inflata* profile 1 but not in the *G. uralensis* profile, which belonged to the SAP (1) and E2F/DP587 (1) gene families; two TFs that were enriched in *G. uralensis* profile 5 but not in *G. uralensis* profile 5, which belonged to the FAR1 (1) and YABBY (1) gene families; and 13 TFs that were enriched in *G. inflata* profile 5 but not in *G. uralensis* profile 5, which belonged to the AP2 (1), GRAS (5), SBP (1), bZIP (2), and Dof (4) gene families (Supplementary Fig. S4). The findings suggested that these 24 TFs play a major role in the salt reaction of both licorice species.

### Quantitative real-time PCR validation

To validate the reliability of our data, we utilized the 18S rRNA gene of *G. inflata* as an internal reference gene. Transcription levels were measured by quantitative real-time PCR, and the results were compared with the RNA-seq data. By linear regression analysis, a coefficient of R^2^ = 0.96154 was obtained for the nine tested transcripts, which indicated the reliability of our data (Supplementary Fig. S5).

## Discussion

### Effects of salt treatment on biomass accumulation of *G. uralensis* and *G. inflata*

Salt stress can profoundly impact plant growth and induce disturbances in plant metabolic processes [32]. Our study demonstrated that the dry weight of the roots and leaves of *G. uralensis* treated with 150 mM NaCl for 15 d and 30 d decreased significantly, which indicated salt stress has adversely affected the growth of *G. uralensis*. However, *G. inflata* appeared to sustain its normal developmental and biomass accumulation even under this saline environment (Fig. 1A, B). Considering that the root is the primary medicinal component of the licorice herb, and its dry weight serves as a crucial metric for licorice yield, obtaining high-quality licorice in saline-alkali soils is imperative for *G. inflata* exhibits a superior capacity to preserve biomass under saline conditions, thus ensuring a robust yield. This is because carbon metabolism can not only maintain the most basic life activities of *G. inflata* but also provides substrates for amino acid and glucose biosynthesis, which usually involves the EMP, TCA cycle and PPP [33–35]. In carbon metabolism, glucose-6-phosphate dehydrogenase (G6PD) facilitates the conversion of glucose-6-phosphate to ribulose 5-phosphate, generating NADPH that integrates with numerous metabolic pathways [14, 36, 37], and the overexpression of citrate synthase in *Arabidopsis* promotes an increase in the citric acid concentration and strengthens its tolerance to salt exposure [35]. Furthermore, the carbon absorbed by plants forms the foundation of their life and metabolic processes [38, 39]. During salinity-induced stress, this carbon assimilation capability, along with the accumulation of biomass, could be compromised in plants [40, 41]. Our findings indicate there could be a small impact of salinity stress on the carbon metabolism of *G. inflata*. We observed that the expression levels of G6PD, citrate synthase, and differentially expressed genes (DEGs) associated with carbon fixation in photosynthetic organisms were conspicuously augmented in *G. inflata*, whereas they were markedly diminished in *G. uralensis* (Fig. 9). Therefore, *G. inflata* can better maintain its necessary energy and substrate supply in saline environments to ensure long-term growth and biomass accumulation. Studies showed that under appropriate salinity levels, there is an enhancement in the metabolic activity of the TCA cycle and glycolytic pathway in sugar beet roots, which helps to maintain cellular vitality [42]. In addition to the biological analysis of key genes G6PD and citrate synthase, other DEGs in carbon metabolism were also identified (Fig. 9). These differentially expressed genes provide more detailed insight into the differences in salt tolerance between the two licorice species.

### Differences in the Na^+^ response between *G. uralensis* and *G. inflata*

Upon the onset of salt stress, plants absorb less water attributed to osmotic stress instigated by the salt, simultaneously manifesting as Na^+^ toxicity [43]. Excessive accumulation of Na^+^ can trigger physiological stress responses in plants, potentially leading to a decline in the quality of secondary metabolites and alterations in their composition [44, 45]. In our study, the content of total flavonoids in *G. uralensis* decreased under salt stress, while *G. inflata* roots can still maintain a stable and high total flavonoid content, despite accumulating more Na^+^ in the roots than *G. uralensis* (Fig. 3). A crucial adaptation of plants to sodium ions is the partitioning of sodium ions among the plant and the environment and the redistribution of sodium ions among different organs, tissues, and cells of the plant [18]. Our research demonstrates that *G. inflata* predominantly accumulates Na^+^ in its roots, in contrast to *G. uralensis* which amasses Na^+^ primarily in its leaves (Fig. 2). Plant species demonstrating superior salt stress resilience typically exhibit pronounced Na^+^ sequestration in the roots, minimizing accumulation in aerial parts [17]. NHX2 regulates the vesicular membrane Na^+^/H^+^ exchanger to compartmentalize Na^+^ in the cytoplasm to vesicles [46]. Additionally, NHX7/SOS1 acts as a plasma membrane Na^+^/H^+^ antiporter, expelling excess intracellular Na^+^ [47, 48]. In our study, 30 d post salt stress compared to *G. uralensis*, *G. inflata* showed marked upregulation of NHX2 and NHX7 (Fig. 10), suggesting enhanced vesicular sequestration and efflux capabilities, which might attenuate cell damage due to salt-induced stress.

Endothelial differentiation is classified into two phases: CS development and suberin lamellae structure formation [31, 49–51]. CS is a lignin-based band structure that acts as a physical barrier in Na^+^ plastid exosome transport [52, 53], impeding the passage of Na^+^ through the mid-column and intercepting and retaining Na^+^ in the root [54]. In lignin precursor biosynthesis, under salt stress, PAL and 4CL are upregulated in the roots of both licorice species (Fig. 11). PAL and 4CL, as raw materials for the synthesis of various compounds, have different regulatory expressions in the two licorice species and could be more widely used to synthesize other active substances, such as flavonoids. The polymerization of lignin precursors occurs in the cell wall and requires transport to the CS formation region (CSD) via a transporter protein before polymerization [55]. ABC transporters are likely to transport proteins for lignin precursors, and ABCG29 can transport H-type lignin precursors [56, 57]. The lignin deposition location depends on the location of PER enzymes, and CASP can recruit PER enzymes that catalyse the oxidation of lignin precursors to form lignin that accumulates in the CSD (Casparian strip membrane domain) [56, 58]. LAC3 plays an important role in the dynamic CSD^W^ of the interface between the primary cell wall and lignin deposition during CS formation, and in precise synergy with CASP, LAC3 regulates lignification in the CSD [59]. Unlike in *G. uralensis*, in *G. inflata* the significant upregulation of the ABC, PER, and CASP families and LAC3 in roots suggests that salt stress may easily promote the translocation of lignin precursors, oxidative deposition, and the development of CS formation (Fig. 11), which enhances the blockade of Na^+^ and allows more Na^+^ to be trapped in the roots, resulting in better protection of the aboveground parts of *G. inflata* against Na^+^ poisoning [60].

Suberin is stored within the interior of the cell wall to develop suberin lamellae, which contain phenolic monomers, aliphatic monomers, and glycerol monomers and play an important role in the response of plants to stress [60–63]. CYP86A1, which regulates the synthesis of aliphatic suberin monomers, catalyses the formation of short-chain ω-hydroxy fatty acids, ranging from C12 to C18 fatty acids, and CYP86B1 catalyses the formation of long-chain ω-hydroxy fatty acids from C22 to C24 fatty acids, which is particularly important during the early deposition of suberin [64, 65], whereas LACS2 efficiently catalyses the synthesis of long-chain fatty acyl-CoA [66]. GLPK participates in catalysing the synthesis of the suberin monomer glycerol [67]. GPAT5 regulates the acyl transfer of the suberin aliphatic monomer fatty acid acyl-coenzyme A to the sn-2 site of G-3-P to generate basal suberin monoacylglycerols, and the aliphatic suberin protein content in *gpat5* mutant *Arabidopsis* roots is reduced by 50% [68]. These genes exhibited increased expression under salt exposure in *G. inflata* at T1, whereas their expression was reduced in *G. uralensis* at T1 (Fig. 11B). This finding indicates that the early biosynthesis of the aliphatic monomer glycerol needed for suberin polymerization may be promoted in *G. inflata* roots and inhibited in *G. uralensis* roots under 150 mM salt treatment. The suberin aliphatic monomers, glycerol, monoacylglycerols, and suberin phenolic monomers are folded, assembled, and deposited to form suberin lamellae [62]. Unlike the trend found in *G. uralensis*, DEGs associated with suberin phenolic monomer biosynthesis under salt treatment (PAL, 4CL, and HHT1) were significantly upregulated in *G. inflata* (Fig. 11B), and the diverse expression of such genes may indirectly contribute to suberin lamellae formation in *G. inflata*. Moreover, transport proteins are still needed for the translocation of suberin monomers from the cytoplasm to the cell wall to form the suberin lamellae, and ABCG20 may be involved in this process. The structural and compositional characteristics of the root suberin lamellae are altered in *Arabidopsis abcg20* mutants [69]. These results suggest that *G. inflata* may promote the extracellular transport of related substances during the initial period of salt exposure to form suberin protective lamellae in the cell wall to control root substance transport and ensure a better response to salt stress.

In our study, *G. inflata* accumulated Na^+^ mainly in the roots, whereas *G. uralensis* accumulated Na^+^ mainly in the leaves, but genes related to long-distance Na^+^ transport were not found to be differentially expressed in the roots of either licorice species. It can be speculated that the different intensities of the action of the endothelial barriers (CS and suberin lamellae) in the roots during Na^+^ transport from the roots to the aboveground parts are perhaps the main cause of the differences in Na^+^ partitioning between these two licorice species like our previous research (Supplementary Fig. S6) [7].

### Differences in osmoregulation between *G. uralensis* and *G. inflata*

The principal defenses against osmotic stress in plants under saline conditions are the uptake, transport, and distribution of inorganic ions as osmoregulatory substances [19]. K^+^, Ca^2+^, and Mg^2+^, are essential nutrients in plants and are also critical for the maintenance of osmotic homeostasis, enzymatic processes, and photosynthesis [70–73]. Unlike in *G. uralensis*, the accumulation of K^+^ in *G. inflata* roots under salt exposure was significantly higher than that in the control (Fig. 2B), which indicates that *G. inflata* can accumulate more K^+^ in roots and can participate in osmoregulatory processes in response to salt exposure and maintain a higher K^+^/Na^+^ level, which ensures better adaptation to salt exposure [14]. A transcriptomic analysis showed that the DEGs that participate in K^+^ release and translocation under salt exposure exhibited significant upregulation in *G. inflata* and significant downregulation in *G. uralensis* (Fig. 10). Among these DEGs, the CHX, HAK5, AKT1, KAT1, and POT genes regulate K^+^ uptake, and the SKOR genes regulate the long-distance transport of K^+^ and release K^+^ to the xylem sap and aboveground parts [74]. Unlike the trend in *G. uralensis*, *G. inflata* under salt exposure not only exhibited increased expression of transporter protein-related genes for increased K^+^ uptake but also exhibited increased long-distance transport of K^+^, which may enable the species to maintain a long-term aboveground K^+^ supply under salt exposure.

We found that the Ca^2+^ content was increased in the roots of both licorice species under salt exposure (Fig. 2C), which could contribute to the maintenance of their cell membrane integrity and cell wall stability under salt exposure. Ca^2+^ plays the role of a second messenger in plants, regulating a complex system of signaling pathways in response to abiotic stress [75–77]. The difference in calcium signal network under salt stress may be indirectly reflected in the difference of salt tolerance between the two kinds of licorice, but more time point verification studies are needed. The expression of the DEGs related to Ca^2+^ uptake and transport was increased markedly in *G. inflata* and decreased in *G. uralensis* during exposure to salt stress (Fig. 10). CCX2, ANN1, and CNGC can regulate Ca^2+^ uptake while CCX2 might inhibit Na^+^ accumulation [78], and MCU and CAX mediate mitochondrial and vesicular Ca^2+^ uptake, respectively. Differential expression of these genes related to Ca^2+^ transporters and calcium signaling promotes Ca^2+^ uptake by cells and organelles in *G. inflata* roots, thereby maintaining a lower osmotic potential and contributing to the maintenance of normal cell swelling.

In addition to regulating inorganic ion uptake in response to salt exposure-induced osmotic stress, plants can also accumulate small-molecule organic solutes (proline, polyamines, betaine, soluble sugars, etc.) to reduce the cellular water potential such that water transport across the membrane occurs in a direction favourable for cell growth [79–81]. The genes regulating proline synthesis (proC, rocD) and degradation (PRODH) were downregulated in the roots of both licorice species (Fig. 12A). Proline may not be a differential osmoregulatory molecule in both licorice species due to restricted synthesis. The synthesis of polyamines helps plants alleviate salt stress-induced osmotic stress, but the long-term accumulation of polyamines inhibits plant growth [23, 82–84]. ADC is involved in the biosynthesis of polyamines, and unlike the trend in *G. uralensis*, ADC was upregulated at T1 and downregulated at T3 in *G. inflata* under salt treatment (Fig. 12A), which may contribute to initial osmoregulation in *G. inflata* and is not negatively affected by long-term accumulation of polyamines. γ-Aminobutyric acid (GABA) can reduce the osmotic water potential in the cytoplasm, improve the water retention performance of cells, and promote plant growth [85]. Aldehyde dehydrogenase (ALDH) is involved in GABA synthesis, and under salt exposure, ALDH was markedly upregulated at T3 in *G. inflata* (Fig. 12A), this may contribute to the synthesis of GABA in *G. inflata* to participate in the reduction of osmotic stress. Studies have shown that the biosynthesis of isoquinoline alkaloids is promoted in many medicinal plants under salt exposure and is beneficial to osmoregulation [86]; for example, betaine, an alkaloid that can stabilize the normal cell volume, increases the free water content of cells and alleviates osmotic stress [87]. The differential upregulation of genes related to isoquinoline alkaloid synthesis (GOT, AOC3, PSMOT1) was more significant under salt exposure in *G. inflata* than in *G. uralensis* (Fig. 12B), implying that this class may also include important differential osmoregulatory substances in the two licorice species.

### Differences in the response to oxidative stress between *G. uralensis* and *G. inflata*

ROS are a “double-edged sword” within living organisms and can act as signaling molecules to activate salt response pathways during the initial periods of salt exposure; however, excessive accumulation of ROS can damage key cell structures and lead to increased permeability of cell membranes [88]. The measurement of the MDA levels in the roots of both licorice species showed that *G. uralensis* suffered more severe oxidative damage under salt exposure (Fig. 1C); however, the Na^+^ content in the roots of *G. uralensis* was lower than that in the leaves, whereas the Na^+^ content in *G. inflata* was mainly concentrated in the roots (Fig. 2A). These results indicate that the roots of *G. inflata* can accumulate a large amount of Na^+^ while suffering less oxidative damage than the roots of *G. uralensis*. Thus, the roots of *G. inflata* have a stronger capacity to scavenge reactive ROS and can better protect themselves from oxidative damage than the roots of *G. uralensis*. The main method used by plants to respond to oxidative stress caused by excessive accumulation of ROS is to synthesize more nonenzymatic antioxidants or activate antioxidant enzymes [22]. The nonenzymatic antioxidant carotenoids can react with membrane lipid peroxidation products to terminate their peroxidation and scavenge ROS through the lutein cycle [89]. The expression of the DEGs regulating lycopene synthesis (crtB, PDS, Z-ISO), δ-carotene synthesis (lcyE), the lutein cycle (crtZ, LUT5, VDE), ABA synthesis (NCED), and degradation (CYP707A) under salt exposure was mainly increased in *G. inflata*, and their expression was decreased in *G. uralensis* (Fig. 13). This finding indicates that salt exposure may promote carotenoid biosynthesis and enhance ROS scavenging in *G. inflata* roots, whereas this effect could be inhibited in *G. uralensis*.

### Differences in flavonoids synthesis between *G. uralensis*and *G. inflata*

Total flavonoids content is usually a key indicator for evaluating the quality of licorice, and flavonoids are important bioactive substances in licorice [90]. Flavonoids have a strong antioxidant capacity, and kaempferol, quercetin, and anthocyanin can improve the salt tolerance of the plant itself [91, 92]. Compared to the control, the total flavonoids content in the roots of *G. inflata* was significantly increased under salt treatment, whereas the in *G. uralensis* was decreased (Fig. 3B). This finding showed that 150 mM salt treatment highly likely inhibited the biosynthesis of flavonoids in *G. uralensis* but was beneficial to the accumulation of flavonoids in *G. inflata* roots. Flavonoid compounds are synthesized as products of the phenylpropane biosynthesis pathway (cinnamoyl-CoA, p-coumaroyl-CoA) [93], and the key enzymes in this synthesis process, PAL and 4CL, were markedly upregulated in *G. inflata* and downregulated in *G. uralensis* (Supplementary Fig. S3), indicating that 150 mM salt treatment may promote the accumulation of cinnamoyl-CoA and p-coumaroyl-CoA in *G. inflata* roots. Chalcone synthase is the first key enzyme in flavonoid biosynthesis and is regulated by CHS; the catalytic production of chalcone is the basis for the synthesis of other flavonoids [94]. HCT uses a variety of acyl-coenzyme A as acyl donors, and the acylated products formed by the catalytic substrate can improve the physicochemical properties of secondary metabolites [95]. Unlike the trend in *G. uralensis*, CHS and HCT were markedly upregulated in *G. inflata* (Fig. 16B), indicating that the biosynthesis of chalcone in *G. inflata* roots was promoted, which facilitated the biosynthesis of other flavonoid compounds. Reduced expression of the anthocyanin reductase (ANR) gene inhibits growth and alters the accumulation of phenolics in silver birch [96], and the FLS-catalysed synthesis of myricetin and kaempferol inhibits metal ion-induced ROS production [97]. In addition, flavonoid 3’-monooxygenase, which is encoded by CYP75B1, catalyses the biosynthesis of quercetin, dihydroquercetin, eriodyctiol luteolin, butin, and dihydrofisetin, and during this process, eriodyctiol luteolin can act as a specific signal to initiate rhizobia symbiosis [98]. These genes were found to be upregulated in *G. inflata* and downregulated in *G. uralensis* (Fig. 14), suggesting that *G. inflata* can synthesize more flavonoids in the roots during salt exposure and regulate its growth and response to antioxidant stress through these flavonoid compounds.

### Differential response of hormone signaling and transcription factors

**Fig. 15:**
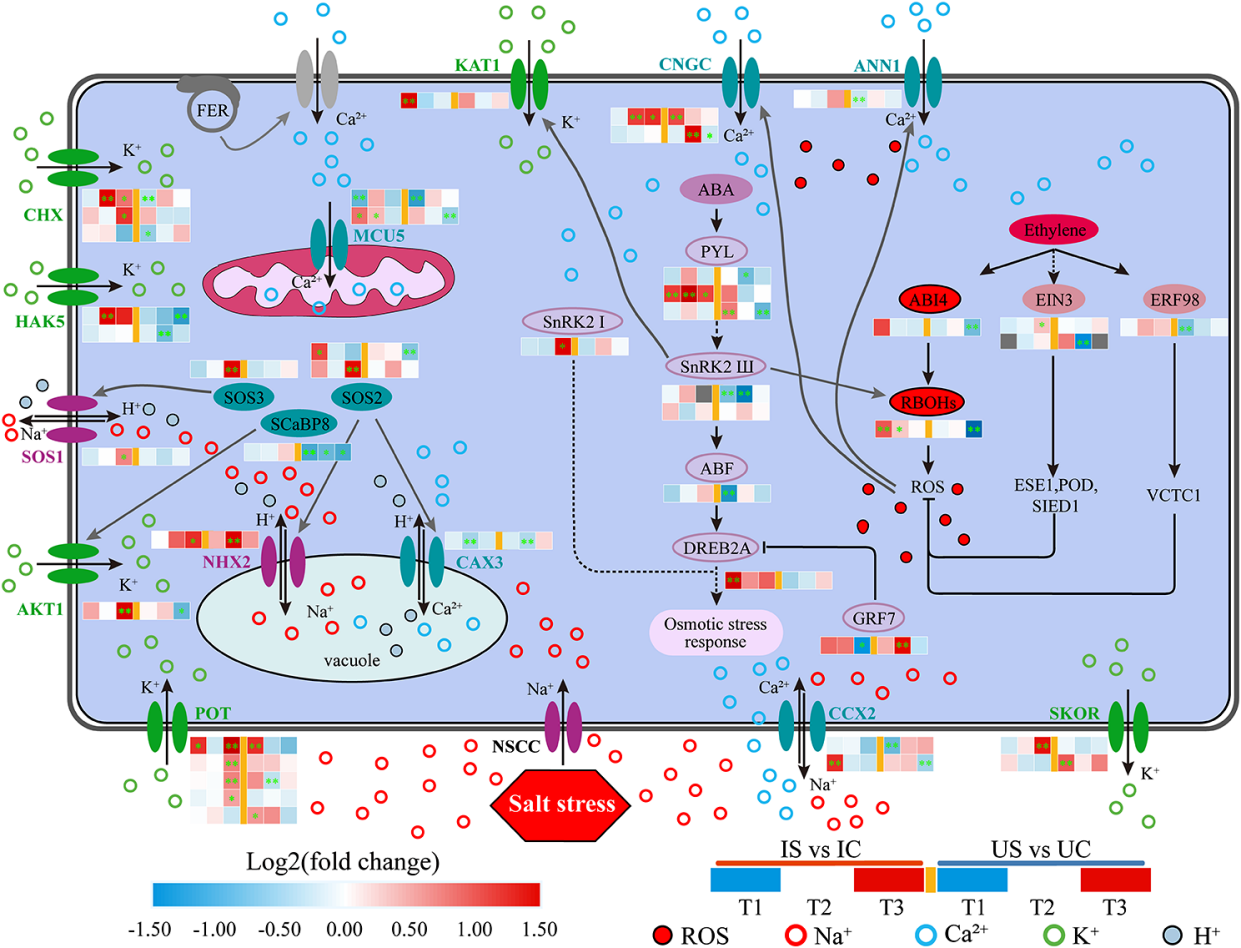
Regulatory network of the salt response of *G. inflata* (I) and *G. uralensis* (I). Significant differences are shown by “*” (*p* < 0.05); highly significant differences are shown by “**” (*p* < 0.01)

The calcium SOS3 gene and the calcium-binding protein 8 (SCaBP8) gene sense the salt-induced increase in Ca^2+^ and promote SOS2 activity through interaction with the SOS2 kinase FISL motif, and SOS2 activates SOS1 by phosphorylating Ser1044 in the C-terminal structural domain of SOS1 [12, 99, 100]. Unlike the trend in *G. uralensis*, SOS3, SOS2, and SCaBP8 were upregulated in *G. inflata* under salt exposure (Fig. 15), indicating that *G. inflata* might have a stronger capacity to secrete excess intracellular Na^+^ to the extracellular compartment through the SOS pathway, which better helps maintain the intracellular K^+^/Na^+^ balance of the plant and regulate the adaptation of the plant to salt exposure. ABA regulates stomatal opening and closing, reduces water loss, and reduces osmotic stress [101, 102]. PYL is the crucial receptor factor in the ABA genetic pathway [103], and the subgroup III SNF1-related protein kinase 2 (subclass III SnRK2) gene is involved in not only the regulation of Ca^2+^ signaling-induced ABA-dependent osmotic stress but also the activation of the cytoplasmic membrane ion channel KAT1, which in turn promotes K^+^ influx, and in the propagation of ROS signals dependent on respiratory burst oxidase homologue proteins (RBOHs) [104, 105]. ABF is the target of the type III SnRK2 TF, and ABF interacts with the DREB2A TF to activate genes and pathways involved in resistance to osmotic stress [106]. In addition, GRF7 represses the expression of DREB2A [107, 108]. Unlike the trend in *G. uralensis*, PYL, SnRK2, ABF, and DREB2A showed upregulated or not markedly different expression, and GRF7 was downregulated in *G. inflata* in this study (Fig. 15). The findings suggested that salt stress may inhibit the response of ABA-dependent type III SnRK2 to osmotic stress in *G. uralensis*, whereas *G. inflata* was not affected by this situation. The ethylene signaling component EIN3 can scavenge excess ROS by activating the expression of genes encoding enzymatic antioxidants such as ESE1, POD, and SOD under salt stress, and the ethylene-inducible factor ERF98 can enhance the salt tolerance of plants by transcriptionally activating the synthesis of the nonenzymatic antioxidant AsA [109]. EIN3 and ERF98 were significantly downregulated in *G. uralensis* after salt exposure (Fig. 15). In contrast to the effect in *G. uralensis*, salt treatment may stimulate the synthesis of enzymatic antioxidants and AsA in *G. inflata* roots through the transduction of ethylene signals, which results in the scavenging of more ROS. ROS signals (RBOHs) can activate ANN1- and CNGC-mediated inwards Ca^2+^ flow and thus produce new Ca^2+^ signal [110]; in addition, their activity is regulated by the upstream TF ABI4, Ca^2+^ signaling, and the ABA pathway [108]. During early salt stress exposure, RBOHs were upregulated in *G. inflata* roots, whereas no significant changes were observed in *G. uralensis*; presumably, RBOHs, as signaling molecules that produce ROS, are activated earlier in *G. inflata*.

TFs regulate gene expression by binding to cis-elements in the promoter regions of target genes [111]. Among others, the WRKY, MYB, bZIP, AP2/ERF, and HSF families have been identified as associated with salt stress, and these families play a central regulatory and molecular switch role in salt stress signaling networks [112]. The production of stress proteins is a positive adaptation of plants to abiotic stress, and heat shock proteins (HSPs) help proteins function properly by preventing irreversible aggregation, denaturation, or loss of the normal folding structure of other proteins in response to environmental stress [113]. In our study, the heat shock TF (HSF) was consistently downregulated in *G. uralensis* under salt exposure (Supplementary Fig. S4), which was not conducive to the effective prevention of protein degradation and misfolding in *G. uralensis* roots. The GRAS TF is cell-autonomous, regulates cortical-endothelial initiator cell division, and stimulates endothelial differentiation [114]. The sustained upregulation of GRAS under salt exposure was found only in *G. inflata* (Supplementary Fig. S4), which may stimulate the formation of an endothelial layer barrier in *G. inflata* roots.

## Conclusion

This study aimed to identify the key pathways and genes associated with the differences in salt tolerance between *G. uralensis* and *G. inflata* at various developmental stages through physiological analysis and transcriptome profiling. Notably, unlike the results obtained for *G. uralensis*, the150 mM salt treatment of *G. inflata* not only maintained the normal dry weight and total flavonoids accumulation but also increased the K^+^ content in the roots while restricting Na^+^ to the roots, ensuring lower MDA levels under high-Na^+^ conditions. Through in-depth quantitative transcriptomic analysis, we revealed the crucial pathways related to the stronger salt tolerance of *G. inflata* compared with *G. uralensis*. These pathways include pathways involved in carbon metabolism, CS formation and development, K^+^ and Ca^2+^ transport, and the biosynthesis of carotenoids and flavonoids, as well as signal transduction pathways and salt-responsive transcription factors. The identification of candidate genes associated with these pathways provides invaluable insights for the breeding of more productive and salt-resistant *G. uralensis* genotypes.

## Materials and methods

### Plant material

*G. inflata* seeds were collected from the *G. inflata* population in Ba Chu, Xinjiang, China, and the seeds of *G. uralensis* were obtained from the experimental station of Shihezi University, which originally obtained these from Hangjin Banner, Inner Mongolia, China. Specimen vouchers of both licorice species seed types were deposited at SHI (Herbarium of Shihezi University). Jiahui Lu formally identified *G. inflata* and *G. uralensis*, and the deposition numbers were “*G. inflata* 12101801” and “*G. uralensis* 201309013”. The names and abbreviations of the plant material are provided for easy reading (Supplementary Fig. S7).

### Plant treatment

All the seeds were subjected to treatment with 85% concentrated H_2_SO_4_ for 30 min and sterilized with 0.1% HgCl_2_ for 10 min. Subsequently, the sterilized seeds were rinsed 3–5 times with sterile water. Afterward, the seeds were sown in small culture pot (with diameter of 10 cm), and the pots were filled with equal volumes of sterilized vermiculite, at a depth of 1.5 cm. These pots were then placed in trays (length of 43 cm, width of 19 cm, and height of 15 cm) that contained 4 L of Hoagland’s solution. The cultures were maintained in a light incubator (GXZ-430D, Ningbo Jiangnan Instrument Factory) with a daytime temperature of 28 °C and a nighttime temperature of 22 °C, and the light intensity was maintained between 280-and 420 µmol·m^− 2^·s^− 1^. Every three days, Hoagland’s solution was replaced, and the precultivation was continued for 21 d. After this period, *G. inflata* and *G. uralensis* seedlings were cultured separately in nutrient solutions containing 0 mmol/L and 150 mmol/L NaCl. Root and leaf samples from each group of plants were collected after 0.5 d, 15 d, and 30 d of treatment for the determination of physiological response indices, transcriptome sequencing, and quantitative PCR material sampling. Sampling for observations of the physiological response and transcriptome sequencing was performed concurrently. Immediately after sampling, the samples were frozen in liquid nitrogen and stored for subsequent sequencing.

### Assessment of the dry weight and MDA levels

MDA levels were measured using Hodges’ method [115]. Fresh root samples (0.5 g) were weighed, chopped, and mixed with 2 mL of 5% trichloroacetic acid (TCA) and a small amount of quartz sand. This mixture was ground into a homogenate, after which 3 mL of TCA was added for further grinding. The homogenate was then centrifuged at 5000 rpm for 10 min. The supernatant obtained was used as the sample extract. Two milliliters of this centrifuged supernatant (with 2 mL of distilled water added for the control) was mixed with 2 mL of 0.67% thiobarbituric acid (TBA) solution and shaken well. The tubes were then placed in a boiling water bath for 20 min (after the appearance of small bubbles in the solution), before being removed and cooled. After further centrifugation at 5000 rpm for 10 min, the absorbance values of the supernatants were measured at 532 nm, 600 nm, and 450 nm wavelengths. In the control tubes, 2 mL of water was used instead of the extract. The MDA concentration was calculated using the following formula: MDA (µmol·L^− 1^) = 6.45(D532-D600)-0.56D450.

### Determination of the Na^+^, K^+^, Ca^2+^ and Mg^2+^ concentration

The seedling roots were washed with distilled water and then immersed in 20 mmol/L Na_2_-EDTA for 15 min to remove ions adhering to the root surface. Samples of the roots and leaves were then heated at 105 °C for 30 min and dried 70 °C to a constant weight. The dried samples were ground into fine powder. For the quantitative analysis, 0.5 g of each sample was accurately weighed using an analytical balance and a horn spoon, with a weight control not exceeding ± 0.03 g for each sample, and the data were recorded. The samples were transferred to clean, dry digestion vessels lined with an inner coating. Using a pipette, 9 mL of high-purity concentrated HNO_3_ and 1 mL of high-purity H_2_O_2_ were added to the digestion vessels, and the mixture was homogenized. The digestion was carried out using a CEM MARS6 microwave digestion system. Following completion of the digestion program, the digestion tubes were placed on a hot plate provided with the instrument, which was set to a temperature of 150 °C, and heated for approximately 2 h to expel the acid. The digest was then diluted to 25 mL in a cleaned volumetric flask and allowed to stand for more than 1 h. The supernatant was transferred to a 10 mL centrifuge tube, and the concentrations of K^+^, Ca^2+^, Na^+^, and Mg^2+^ in the root and leaf samples were determined using an inductively coupled plasma optical emission spectrometer (ICAP-6300).

### Determination of the total flavonoids content

The roots and leaves of the plants were dried in an oven at 70 °C until a constant weight was achieved, and the plants were then ground into a fine powder. Precisely 0.5 g of the sample powder was weighed and placed into a 10 mL centrifuge tube, and 5 mL of methanol was then added to the tube. The mixture was subjected to ultrasonic extraction for 70 min. Subsequently, the was centrifuged at 5000 r/min for 15 min, and the supernatant was then collected as the sample solution. Briefly, 3 mg of the standard compound glycyrrhizin was weighed and dissolved in 3 mL of methanol to prepare the standard storage solution. Aliquots of 0 µL, 50 µL, 100 µL, 200 µL, and 400 µL were collected from the standard solution into centrifuge tubes, to which 1 mL of methanol and 250 µL of 10% potassium hydroxide were then added. The mixture was allowed to develop color for 5 min, diluted to 5 mL with methanol and thoroughly mixed. The absorbance was measured at a wavelength of 334 nm to plot the standard curve against the corresponding concentrations of 0 µg/mL, 3.35 µg/mL, 6.89 µg/mL, 14.243 µg/mL, and 24.92 µg/mL. The regression equation was Y = 0.0124X + 0.146 with an R^2^ value of 0.9976, indicating a good linear relationship in the range of 0 to 24.92 µg/mL. For the sample analysis, 100 µL of the prepared sample solution was mixed with 250 µL of 10% potassium hydroxide and 1 mL of methanol. After a color development period of 5 min, the volume was increased to 5 mL with methanol, and the solution was thoroughly mixed. The absorbance at 334 nm was measured to determine the total flavonoids content, which was expressed as a percentage of the dry weight of the extract. Glycyrrhizin (090708) was purchased from Shanghai Winherb Medical Science Co.Ltd.

### Total RNA isolation and Illumina sequencing

Total RNA was extracted from the root tissues (0.5 d, 15 d and 30 d) of the salt-treated and control groups of *G. inflata* and *G. uralensis* plants using TRIzol reagent. Three biological replicates were processed per treatment to obtain a total of 36 root samples for RNA sequencing, each of which was approximately 0.1 g. RNA decomposition was checked with a 1% agarose gel, after which the purity of the RNA was assessed. The RNA concentration was determined with a Qubit® RNA, and the RNA integrity was assessed with an RNA Nano 6000 Analysis Kit (USA). The generation of sequencing libraries was performed with an NEBNext® Ultra™ RNA kit (USA), the library fragments were purified using the AMPure XP system, and cDNA fragments were then screened and subjected to PCR amplification. The samples were purified to evaluate their quality. Downstream sequencing data in FASTQ format were analyzed using Perl scripts to obtain high-quality clean sequences (GC content, Q20, Q30 assessment), and HISAT2 was used to build an index of the licorice genome and compare its data [116]. .

### Differentially expressed gene analysis

The gene reads were subsequently converted using HTSeq and expression matrices with FPKM values were obtained. DEG analysis can be performed comparing two different conditions (IST1 vs. ICT1, IST2 vs. ICT2, IST3 vs. ICT3, UST1 vs. UCT1, UST2 vs. UCT2, UST3 vs. UCT3) using DESeq R. The p-value is then adjusted [117]. For WGCNA and STEM analysis, the screening criteria for DEGs (16,086) were q < 0.05 and |log2FoldChange| > 1. In the Mfuzz analysis, the screening criteria for DEGs (34,263) were q < 0.05 and FPKM > 1 to enrich path information in fuzzy clustering. Fuzzy clustering of the DEGs after using the Mfuzz package from R and selecting 5 trends. WGCNA was performed using the WGCNA package from R for FPKM of all DEGs, soft power selected 9, and DEGs were filtered and screened. The screened DEGs were again subjected to GO and KEGG analysis, and a co-expression network diagram was generated. The STEM analysis given the log2 (fold change) values of the respective salt-responsive DEGs of *G. inflata* (IST1 vs. ICT1, IST2 vs. ICT2, IST3 vs. ICT3) and *G. uralensis* (UST1 vs. UCT1, UST2 vs. UCT2, UST3 vs. UCT3) allowed the analysis of the differences in the salt-responsive gene expression trends of the two licorices species. Each Profile obtained by STEM was used for GO and KEGG enrichment assays (GOseq R, KOBAS) while correcting for GO terms, and KEGG pathway enrichment (*p* < 0.05) [118].

### Quantitative real-time PCR

Total RNA was extracted from 0.1 g samples of root tissues from both *G. inflata* and *G. uralensis* in the salt-treated groups (0.5 d, 15 d, 30 d) and control groups (0.5 d, 15 d, 30 d) and was used as a template for analysis. The RNA concentration and purity were assessed using a NanoDrop UV spectrophotometry (NanoDrop2000, Thermo), and RNA extraction quality was measured by 2% agarose gel electrophoresis. Quasi-reverse transcription of RNA samples was performed using a gene synthesis kit (Thermo Fisher Scientific, USA). PCR tubes (0.2 mL) were prepared, and 3 tubes were prepared for PCR amplification of each reverse transcription product. In addition, reaction systems for the reverse transcription of RNA into cDNA (Supplementary Table S3), reaction temperature and reaction time (Supplementary Table S4), and reaction systems for PCR amplification (Supplementary Table S5) were provided. qPCR was performed using real-time PCR system (ABI, Stepone plus, USA). Reaction procedure: pre-denaturation 95 °C, 5 min; repeat for 20 s,55 °C, 20 s, 72 °C, 20 s, number of cycles 40; temperature 72 °C, 5 min. According to Livak KJ & Schmittgen TD (2001), real-time quantitative PCR and 2-ΔΔCT were used to analyze relevant gene expression data [119]. The specific primers used for qRT‒PCR amplification of the genes (Supplementary Table S6) were designed by Primer Premier 6.0 (PREMIER Biosoft, CA, USA), and the 18 S RNA gene of *G. inflata* was used as the reference gene.

### Statistical analysis

The dry weight, MDA content, ion content, and total flavonoids content (3 biological replicates for each level) were analysed by ANOVA using R language software and Microsoft Excel. Significant differences were assessed using Tukey’s test (Tukey HSD function of the stats package). All the data are expressed as the means ± standard errors. The licorice reference genome was obtained from github.com/BioproductivityInformaticsResearchTeam/Glycyrrhiza_uralensis_genome.

### Electronic supplementary material

Below is the link to the electronic supplementary material.

Supplementary Material 1

Supplementary Material 2

Supplementary Material 3

Supplementary Material 4

Supplementary Material 5

Supplementary Material 6

Supplementary Material 7

Supplementary Material 8

Supplementary Material 9

Supplementary Material 10

Supplementary Material 11

Supplementary Material 12

Supplementary Material 13

## Data Availability

The raw RNA-seq data (BioProject: PRJNA977447) are available at:https://www.ncbi.nlm.nih.gov/bioproject/PRJNA977447.

## Abbreviations

PAL: Phenylalanine ammonia-lyase
4CL: 4-coumarate-CoA ligase
CHS: Chalcone synthase
HCT: Hydroxycinnamoyl
FLS: Flavonol synthase
ANR: Anthocyanidin reductase
CYP75B1: Flavonoid 3’-monooxygenase
GRAS: Scarecrow-like protein
bHLH: Basic helix-loop-helix
KEGG: Kyoto encyclopedia of genes and genomes
GO: Gene Ontology
KEGG: Kyoto Encyclopedia of Genes and Genomes
STEM: Short Time-series Expression Miner
Mfuzz: Fuzzy C-Means Clustering
