# Supplementary material for: Integrative physiology and transcriptome reveal salt-tolerance differences between two licorice species: Ion transport, Casparian strip formation and flavonoids biosynthesis
Source: BMC Plant Biol. 2024 Apr 11;24:272. doi: 10.1186/s12870-024-04911-1 (PMC11007891; doi:10.1186/s12870-024-04911-1)
Supplement: Supplementary file 1 — Supplementary Material 1 [file 12870_2024_4911_MOESM1_ESM.docx]

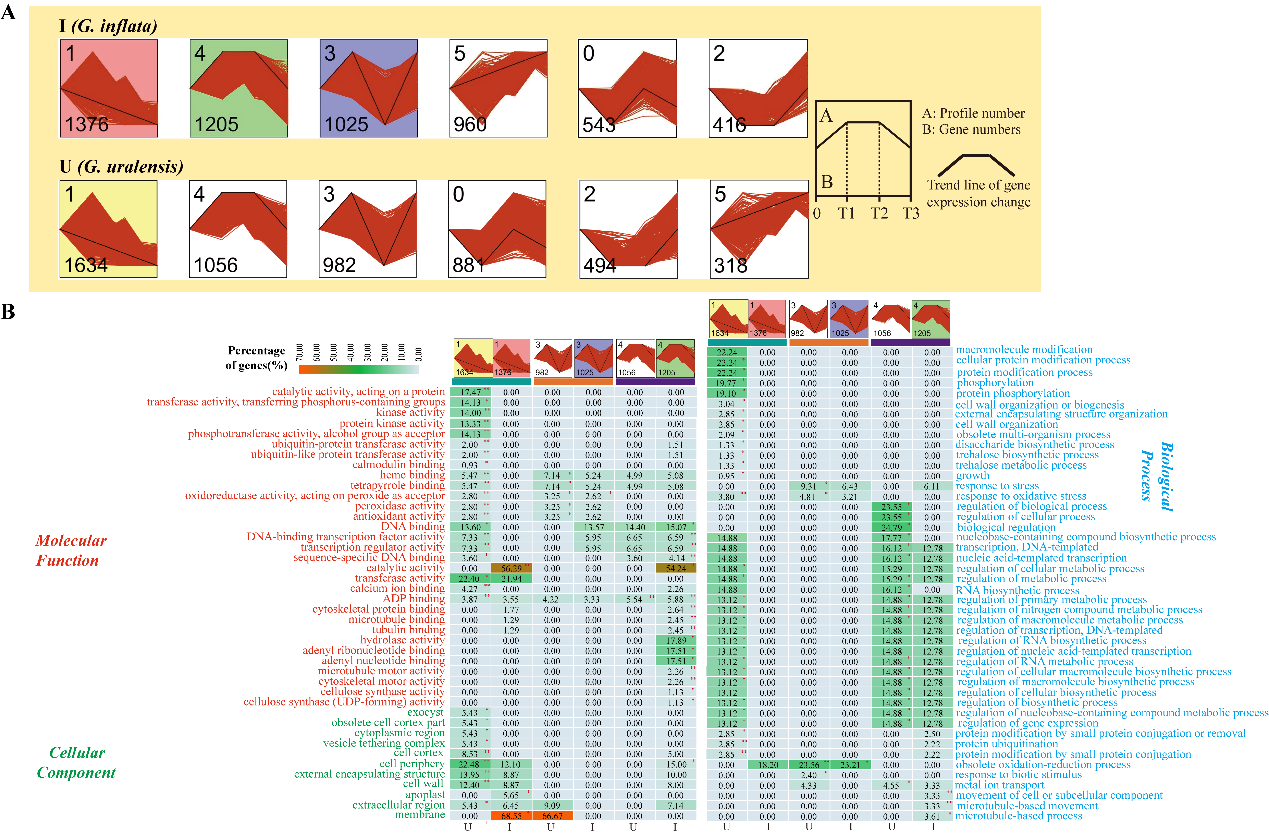


Supplementary Figure. S1. (**A**) Analysis of differential gene expression trends in the two licorice species in different salt stress stages. (**B**) Analysis of the GO enrichment trends of the *G. inflata* (I) and *G. uralensis* (U) DEGs in different salt exposure stages. Significant differences are shown by "*" (*p* < 0.05); highly significant differences are shown by "**" (*p* < 0.01). The value represents gene percentage numbers of annotated DEGs for the GO term/total number of annotated DEGs.
