# Supplementary material for: Integrative physiology and transcriptome reveal salt-tolerance differences between two licorice species: Ion transport, Casparian strip formation and flavonoids biosynthesis
Source: BMC Plant Biol. 2024 Apr 11;24:272. doi: 10.1186/s12870-024-04911-1 (PMC11007891; doi:10.1186/s12870-024-04911-1)
Supplement: Supplementary file 2 — Supplementary Material 2 [file 12870_2024_4911_MOESM2_ESM.docx]

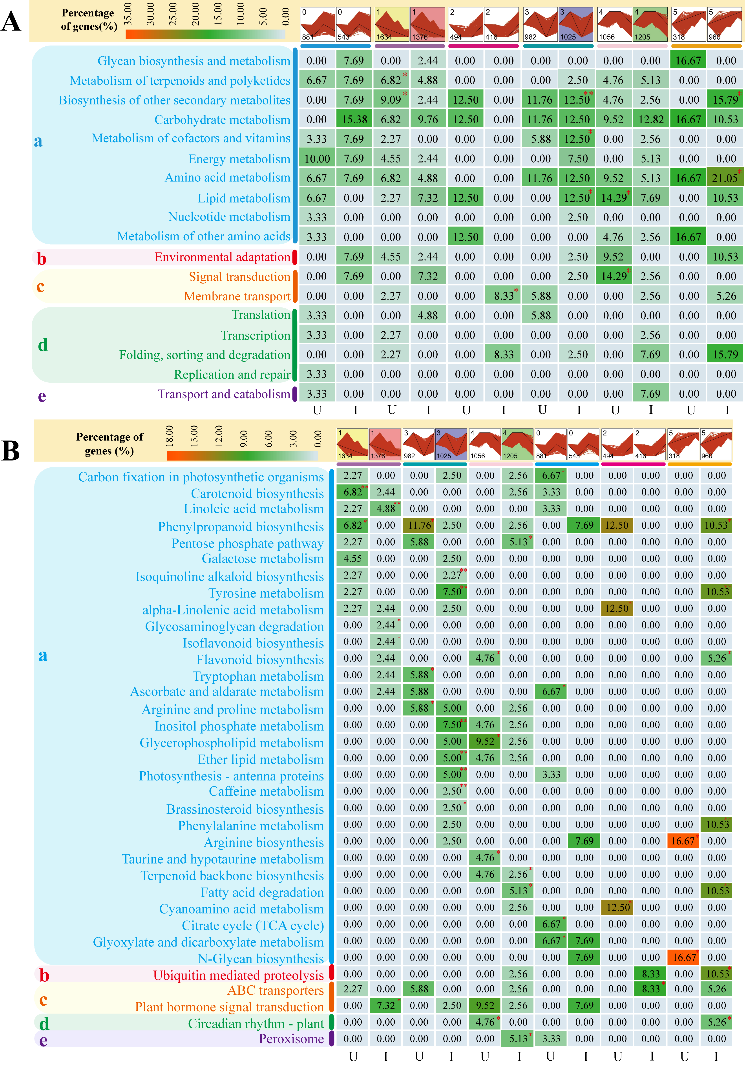


Supplementary Figure. S2. Analysis of the KEGG enrichment trends of the DEGs in the two licorice species in different salt stress stages. (**A**) Metabolism [a], organismal systems [b], environmental information processing [b], genetic information processing [d], and cellular processes [e]. Significant differences are shown by "*" (*p* < 0.05); highly significant differences are shown by "**" (*p* < 0.01). The value represents gene percentage numbers of annotated DEGs for the KEGG term/total number of annotated DEGs. (**B**) Detailed annotations for five class KEGG analysis.
