# Supplementary material for: Integrative physiology and transcriptome reveal salt-tolerance differences between two licorice species: Ion transport, Casparian strip formation and flavonoids biosynthesis
Source: BMC Plant Biol. 2024 Apr 11;24:272. doi: 10.1186/s12870-024-04911-1 (PMC11007891; doi:10.1186/s12870-024-04911-1)
Supplement: Supplementary file 3 — Supplementary Material 3 [file 12870_2024_4911_MOESM3_ESM.docx]

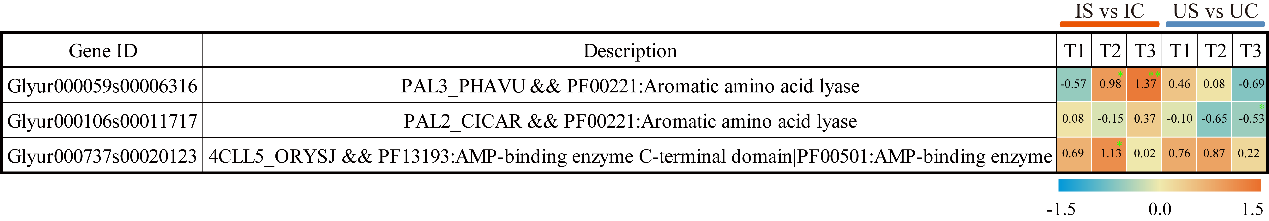


Supplementary Figure. S3. Heatmap of DEGs related to precursors of the flavonoids in *G. inflata* (I) and *G. uralensis* (U).
