# Supplementary material for: Integrative physiology and transcriptome reveal salt-tolerance differences between two licorice species: Ion transport, Casparian strip formation and flavonoids biosynthesis
Source: BMC Plant Biol. 2024 Apr 11;24:272. doi: 10.1186/s12870-024-04911-1 (PMC11007891; doi:10.1186/s12870-024-04911-1)
Supplement: Supplementary file 4 — Supplementary Material 4 [file 12870_2024_4911_MOESM4_ESM.docx]

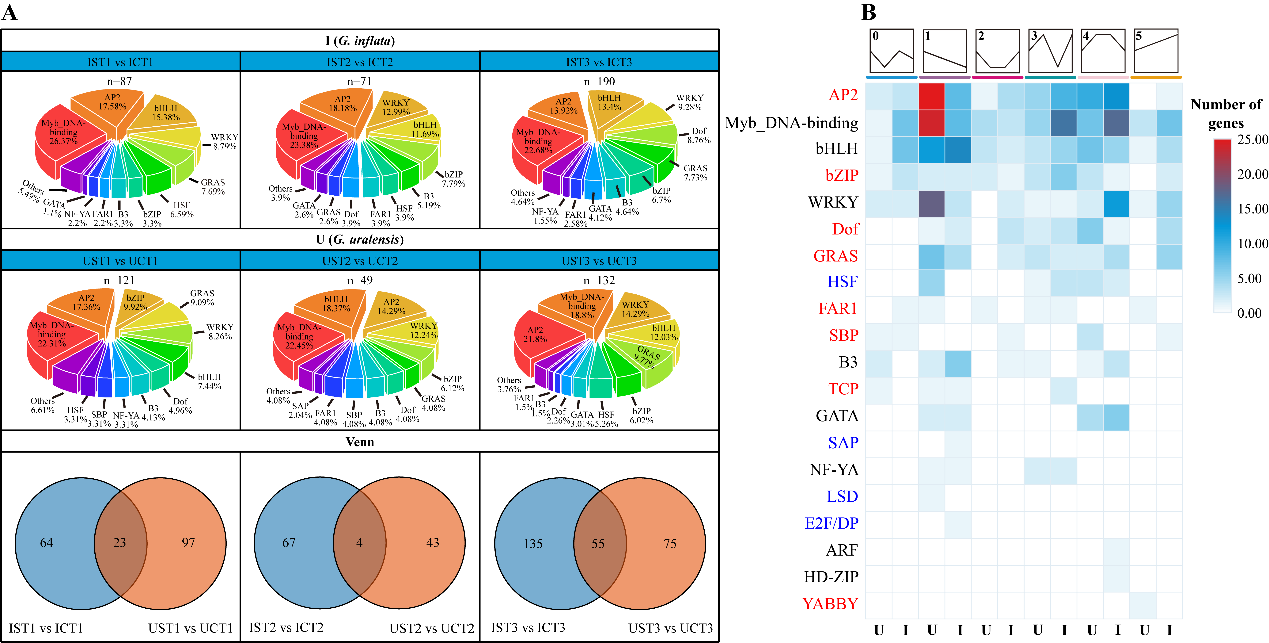


Supplementary Figure. S4. (**A**) Differential expression of transcription factors (TFs) in the two licorice species under salt exposure. (**B**) Different expression trends of transcription factors (TFs) of the two licorice species in different salt exposure periods.
