# Supplementary material for: Integrative physiology and transcriptome reveal salt-tolerance differences between two licorice species: Ion transport, Casparian strip formation and flavonoids biosynthesis
Source: BMC Plant Biol. 2024 Apr 11;24:272. doi: 10.1186/s12870-024-04911-1 (PMC11007891; doi:10.1186/s12870-024-04911-1)
Supplement: Supplementary file 5 — Supplementary Material 5 [file 12870_2024_4911_MOESM5_ESM.docx]

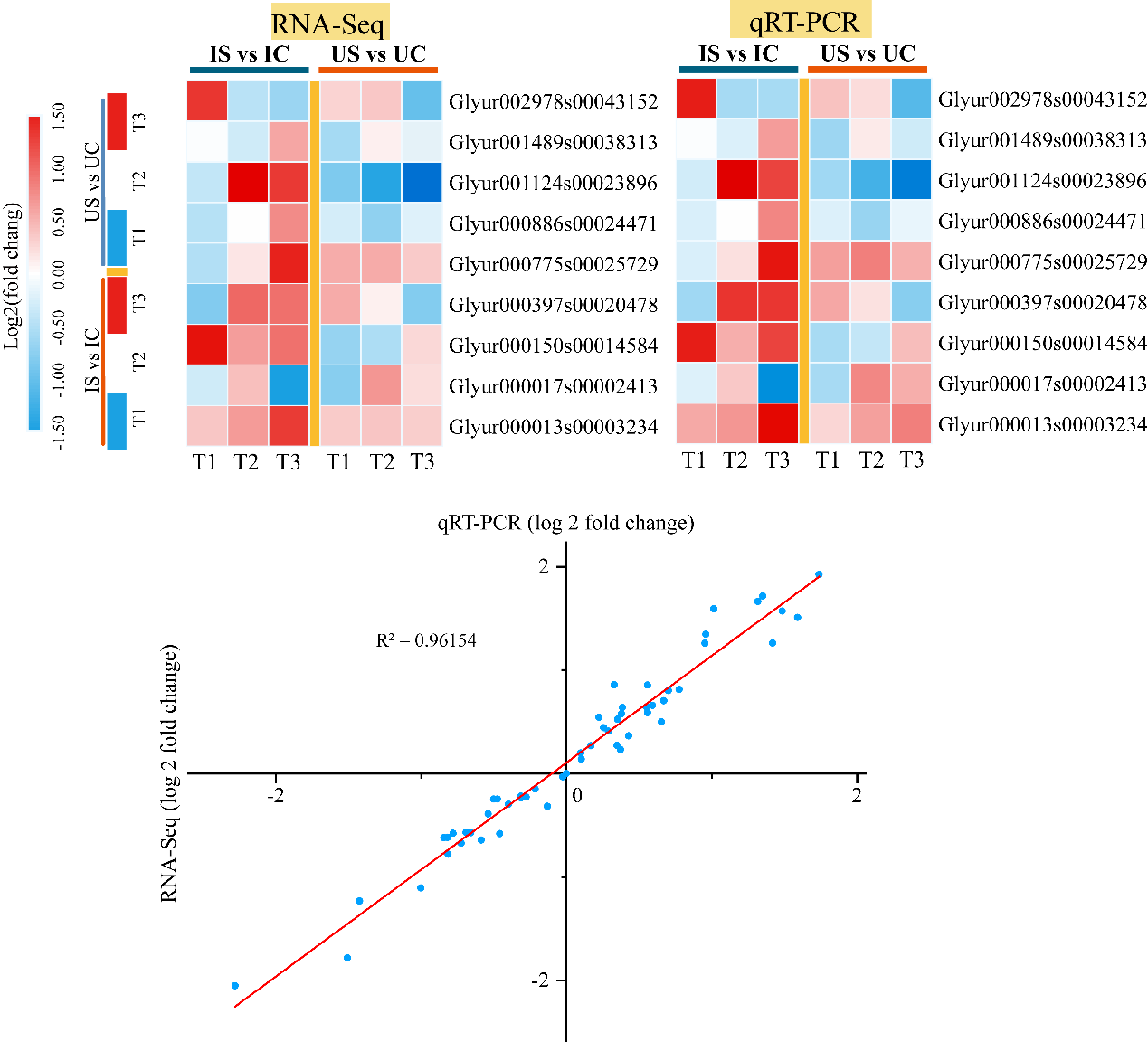


Supplementary Figure. S5. Expression verification of DEGs. The heatmap shows the differential expression of the transcriptome and experimental group of 9 genes (log_2_fold change), and the line graph is the correlation analysis of the qRT-PCR data and the RNA sequencing data.
