# Supplementary material for: Integrative physiology and transcriptome reveal salt-tolerance differences between two licorice species: Ion transport, Casparian strip formation and flavonoids biosynthesis
Source: BMC Plant Biol. 2024 Apr 11;24:272. doi: 10.1186/s12870-024-04911-1 (PMC11007891; doi:10.1186/s12870-024-04911-1)
Supplement: Supplementary file 6 — Supplementary Material 6 [file 12870_2024_4911_MOESM6_ESM.docx]

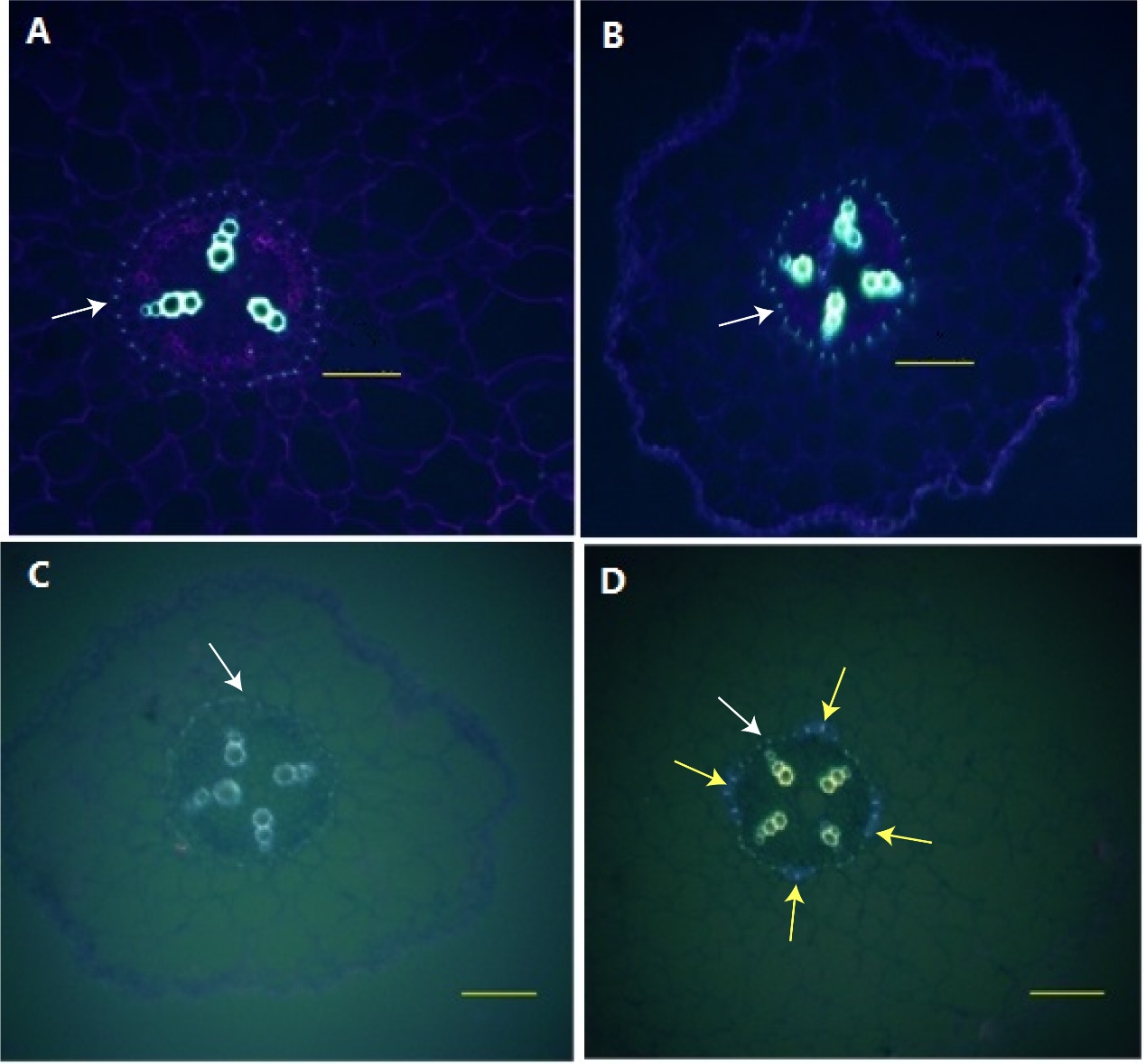


Supplementary Figure. S6. Development of Casparian strip (white arrow) and suberin (yellow arrow) in the endodermis of *G. uralensis* (A, B) and *G. inflata* (C, D) at 20-25 mm from the root tip. Bars=50µm. *Note:* A and C were treated with 0 mM NaCl while B and D were treated with 150 mM NaCl.
