# Supplementary figures and images for: Integrative physiology and transcriptome reveal salt-tolerance differences between two licorice species: Ion transport, Casparian strip formation and flavonoids biosynthesis
Source: BMC Plant Biol. 2024 Apr 11;24:272. doi: 10.1186/s12870-024-04911-1 (PMC11007891; doi:10.1186/s12870-024-04911-1)

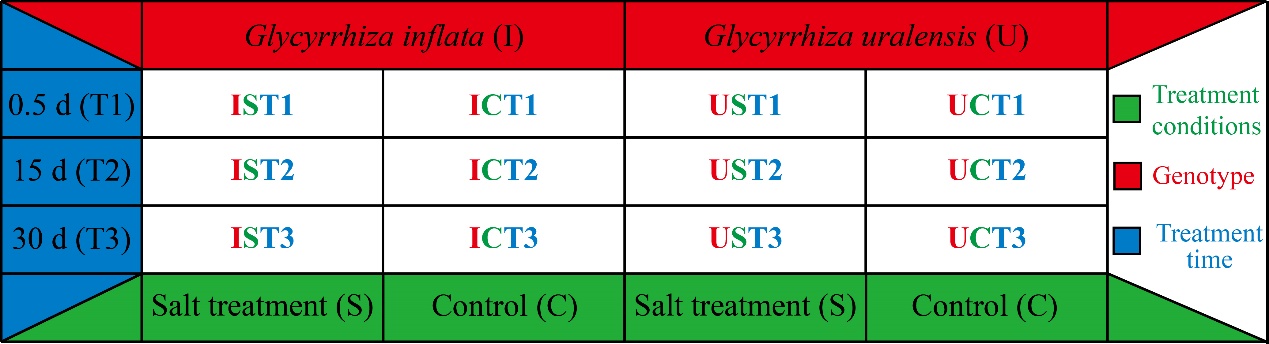


Supplementary Figure. S7. Nomenclature and grouping of licorice experimental materials.

Supplement: Supplementary file 7 — Supplementary Material 7 [file 12870_2024_4911_MOESM7_ESM.docx]
